# Regulation of Pom cluster dynamics in *Myxococcus xanthus*

**DOI:** 10.1371/journal.pcbi.1006358

**Published:** 2018-08-13

**Authors:** Silke Bergeler, Erwin Frey

**Affiliations:** Arnold Sommerfeld Center for Theoretical Physics and Center for NanoScience, Department of Physics, Ludwig-Maximilians-Universität München, Munich, Germany; Rice University, UNITED STATES

## Abstract

Precise positioning of the cell division site is essential for the correct segregation of the genetic material into the two daughter cells. In the bacterium *Myxococcus xanthus*, the proteins PomX and PomY form a cluster on the chromosome that performs a biased random walk to midcell and positively regulates cell division there. PomZ, an ATPase, is necessary for tethering of the cluster to the nucleoid and regulates its movement towards midcell. It has remained unclear how the cluster dynamics change when the biochemical parameters, such as the attachment rates of PomZ dimers to the nucleoid and the cluster, the ATP hydrolysis rate of PomZ or the mobility of PomZ interacting with the nucleoid and cluster, are varied. To answer these questions, we investigate a one-dimensional model that includes the nucleoid, the Pom cluster and PomZ proteins. We find that a mechanism based on the diffusive PomZ fluxes on the nucleoid into the cluster can explain the latter’s midnucleoid localization for a broad parameter range. Furthermore, there is an ATP hydrolysis rate that minimizes the time the cluster needs to reach midnucleoid. If the dynamics of PomZ on the nucleoid is slow relative to the cluster’s velocity, we observe oscillatory cluster movements around midnucleoid. To understand midnucleoid localization, we developed a semi-analytical approach that dissects the net movement of the cluster into its components: the difference in PomZ fluxes into the cluster from either side, the force exerted by a single PomZ dimer on the cluster and the effective friction coefficient of the cluster. Importantly, we predict that the Pom cluster oscillates around midnucleoid if the diffusivity of PomZ on the nucleoid is reduced. A similar approach to that applied here may also prove useful for cargo localization in ParAB*S* systems.

## Author summary

In order for the rod-shaped bacterium *M. xanthus* to reproduce, its genetic content must be duplicated, distributed equally to the two cell halves and then the cell must divide precisely at midcell. Three proteins, called PomX, PomY and PomZ, are important for the localization of the cell division site at midcell. PomX and PomY form a cluster and PomZ tethers this cluster to the bacterial DNA or nucleoid (region containing the chromosomal DNA) and is important for the movement of the cluster from the nucleoid pole towards midcell. We are interested in the question how the cluster trajectories change when the PomZ dynamics is varied. To address this question we investigate a previously developed mathematical model that incorporates the nucleoid, the cluster and PomZ. We simulated the cluster trajectories for different model parameters, such as different diffusion constants of PomZ on the nucleoid. Interestingly, when PomZ diffuses slowly on the nucleoid, we observed oscillatory cluster movements around midcell. Our results provide general insights into intracellular positioning of proteins.

## Introduction

The formation of protein patterns and the intracellular positioning of proteins is a major prerequisite for many important processes in bacterial cells, such as cell division. In order to maintain the genetic content of the bacterial cell, the chromosome (nucleoid) is duplicated during the cell cycle and must be segregated into the two cell halves prior to cell division. The future division site is defined by the FtsZ ring, which forms at midcell and recruits the cytokinetic machinery. Interestingly, FtsZ is highly conserved in bacteria, while the protein systems responsible for the positioning of the FtsZ ring, and with it the cell division site, are not [1–3].

Recently, Schumacher et al. identified a set of proteins, called PomX, PomY and PomZ, in *Myxococcus xanthus* cells that are important for midcell localization and formation of the FtsZ ring [4–6]. PomZ is an ATPase, which belongs to the family of ParA / MinD ATPases [3]. It binds non-specifically to DNA in its dimeric, ATP-bound state, and its activity is stimulated by interactions with PomX, PomY and DNA. PomX and PomY form a single cluster, which is tethered to the nucleoid via PomZ dimers bound to the chromosome. Starting from an off-center position near one nucleoid pole, the cluster moves towards midnucleoid, coinciding with midcell [5]. When the cluster has reached midcell, the FtsZ ring forms there and the cell divides. During cell division, the cluster splits into two halves, such that each half is located at one pole of the nucleoids in the daughter cells, and the same cycle repeats. Notably, the Pom proteins localize to midcell before FtsZ and also in the absence of FtsZ [4, 5].

The mechanism underlying midcell localization of the FtsZ ring is well understood in *Escherichia coli* cells [7–21]. Here, Min proteins (MinC, MinD and MinE) guide the formation of the FtsZ ring at midcell. Importantly, the system for midcell localization in *E. coli* and *M. xanthus* differ substantially, even though both systems contain an ATPase (PomZ and MinD, respectively) and perform the same task in the cell, i.e. midcell sensing. First, the scaffold to which the ATP-bound ATPase binds is different: MinD binds to the cell membrane and PomZ to the bacterial nucleoid in the cytoplasm. Second, MinD-bound MinC inhibits [22], whereas the Pom cluster promotes FtsZ ring formation at midcell [5]. Finally, the observed protein patterns differ: the Pom proteins colocalize in a cluster that moves towards midcell, while the Min proteins, which do not form a cluster, oscillate from pole to pole [22, 23].

Conversely, the Pom system is mechanistically more similar to plasmid and chromosome segregation systems that involve a ParAB*S* system. Like the Pom system, plasmid and chromosome segregation systems make use of an ATPase that shuttles one or several cargoes (such as a plasmid, a partition complex or a protein cluster) along the nucleoid. To ensure the equal distribution of low-copy number plasmids to the daughter cells, they are tethered to the nucleoid and positioned at equal distances along the nucleoid by ParAB*S* systems [24–26]. A ParAB*S* system consists of the proteins ParA and ParB, and a DNA sequence, *parS*. ParA proteins are ATPases, which bind non-specifically to DNA as ATP-bound dimers [27–29]. Their ATPase activity is stimulated in the presence of ParB [30–32], which binds to the *parS* sequence on the chromosome (to form the partition complex) or on the plasmid [3]. Besides plasmid and chromosome segregation [33], ParAB*S* systems are also important for the positioning of cellular components (e.g. chemotactic clusters or carboxysomes) [34, 35]. Several different cargo dynamics involving ParAB*S* systems have been observed. For one cargo these localization patterns include, among others, midcell localization [26, 36, 37], oscillatory movement of ParA and its cargo [24, 25, 38] as well as movement from one cell pole to the other [39]. Multiple cargoes are found to equidistantly position along the nucleoid [24–26, 36, 37].

To account for the dynamics observed in Par systems, various mechanisms have been proposed. Some models rely on ParA filament formation [24, 30, 33, 40–43], others challenge this assumption in *in vivo* systems [39, 44, 45]. A diffusion-ratchet mechanism for the movement of ParB-coated beads *in vitro* and DNA segregation *in vivo* has been introduced [32, 45–49]. Based on the observation that DNA has elastic properties [39, 50], a DNA-relay mechanism for the movement of the partition complex was proposed [39, 51]. Here, the force exerted on the cargo is attributed to the elastic properties of the chromosome. Ietswaart et al. observed that if a plasmid is located off-center on the nucleoid, the ParA flux from the left and right sides of the plasmid differ [36]. Based on this idea, they proposed a model that produces equal plasmid spacing over the nucleoid as long as the plasmid moves in the direction of the higher ParA concentration [36]. Additionally, models based on reaction-diffusion equations for Par protein dynamics, have been introduced [52–55].

In order to account for the experimental observations in *M. xanthus* cells, we have proposed a model for midcell localization [5] that is inspired by, but also differs from previous models for Par systems (see S1 Text). The key experimental observations for the Pom system are: First, PomZ accumulates at the cluster consisting of PomX and PomY proteins, which is in contrast to a low ParA density at plasmids / the partition complex [24, 47]. Conversely, there are also positioning systems that show an accumulation of the ATPase at the cargo [34, 56], which resembles the observations for the Pom system. Second, the cluster is relatively large (0.7 μm in length, [5]) compared to plasmids / partition complexes (about 0.1 μm in length, [39]). Third, the PomZ proteins diffuse quickly on the nucleoid compared to a slowly moving Pom cluster [5]. Finally, fluorescence micrographs of *M. xanthus* cells do not show a clear depletion zone in PomZ in the wake of the cluster, in contrast to observations for Par systems [24, 38, 47]. The latter observation can be explained by the fast PomZ dynamics on the nucleoid. Our model suggests a positioning mechanism that relies on the biasing of fluxes of PomZ dimers on the nucleoid, similar to the equipositioning mechanism proposed by Ietswaart et al. [36]. With this model we were able to reproduce midnucleoid localization with physiologically relevant parameters [5], but it remained unclear how the movement of the cluster changes when the rates of the key biological processes involved are varied.

Here, we investigate the robustness of Pom cluster dynamics in our model with respect to different parameters, so that our model can be tested by experimentally examining this robustness. Interestingly, we observe that there exists an intermediate ATP hydrolysis rate that minimizes the time the clusters need to reach midnucleoid. Furthermore, we find that fast diffusion of PomZ dimers on the cluster accelerates the movement of the cluster towards midnucleoid. To gain a better understanding of the cluster dynamics observed in the *in silico* parameter sweeps, we investigate how PomZ dimers generate a net force on the cluster in our model. For the case where the PomZ gradient builds up faster than the velocity of cluster movement, we derive a semi-analytical approximation for the average cluster trajectory, which dissects the generation of a net force into two parts: the difference between the diffusive PomZ fluxes into the cluster from either side, and the force exerted by a single PomZ dimer during its interaction with the cluster. This net force can account for the movement of the cluster to midnucleoid. In contrast, when the PomZ dimers diffuse slowly on the nucleoid, we observe oscillatory cluster movement.

## Results

### Stochastic model

**Fig 1:**
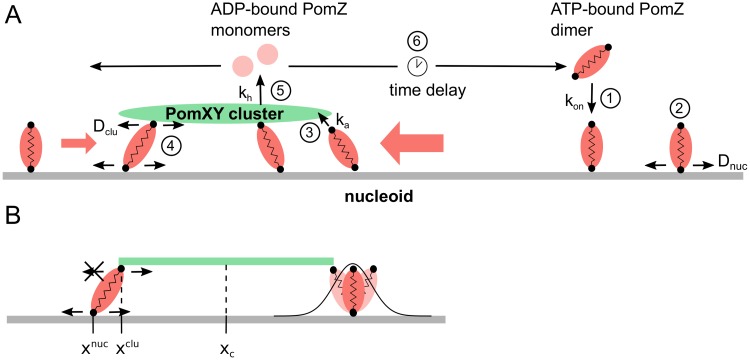
Flux-based model for midnucleoid positioning. (A) In our mathematical model, ATP-bound PomZ dimers can attach to the nucleoid (1) and then diffuse along it (2). The elasticity of the chromosome and the PomZ dimers is effectively included by modelling the PomZ dimers as springs. A nucleoid-bound PomZ dimer has a free binding site available to bind to the PomXY cluster (3). When also bound to the PomXY cluster, a PomZ dimer can diffuse both on the cluster and on the nucleoid (4). The interaction of PomZ with the PomXY cluster (and DNA) leads to a stimulation of the ATPase activity of PomZ, which in turn causes a conformational change in the ATP-bound PomZ dimer and the release of two ADP-bound monomers into the cytosol (5). ADP-bound PomZ monomers must exchange ADP for ATP and form dimers before they can bind to the nucleoid again (these processes are not explicitly included in the model). Hence, there is a delay between release of the inactive, ADP-bound form and reconstitution of the active, ATP-bound form (6). (B) Details of the PomZ interactions with the PomXY cluster. Not only PomZ dimers with a nucleoid binding site below the PomXY cluster, but also PomZ dimers outside of the cluster region can attach to the cluster, in a stretched configuration. The edges of the PomXY cluster are reflecting boundary conditions for the movement of PomZ’s cluster binding site (indicated by the crossed arrow).

We employ a stochastic lattice gas model, introduced in [5], to understand the dynamics of the PomXY cluster, i.e. the cluster consisting of PomX and PomY proteins, in *M. xanthus* bacterial cells [5]. In this model, both the nucleoid and the PomXY cluster are reduced to one-dimensional lattices of length *L* and *L*_*c*_, respectively (Fig 1). Our model consists of two parts: first, the PomZ dynamics, and second the PomXY cluster movement due to its interactions with PomZ. We first describe the PomZ dynamics in the next paragraph.

PomZ can occur in different configurations: it can be bound to ADP or ATP and in the latter case, form dimers. In our model we incorporate only the ATP-bound dimeric form of PomZ explicitly. We model the PomZ dimers effectively as springs with spring stiffness *k* to account for the elastic properties of the chromosome and the PomZ dimers. Each PomZ dimer spring has two binding sites, one which connects to the nucleoid, and one which connects to the PomXY cluster. ATP-bound PomZ dimers can bind with the first binding site to the nucleoid with rate *k*_on_ (Fig 1A(1)), except where the PomXY cluster is located, and diffuse on the nucleoid with diffusion coefficient *D*_nuc_ (Fig 1A(2)). Because of thermal fluctuations, the relative position of the second binding site, which enables PomZ to bind to the PomXY cluster, is distributed according to a Boltzmann distribution with the energy of the spring. Therefore, PomZ dimers can attach to the PomXY cluster even if their nucleoid binding sites are not directly below the cluster (Fig 1A(3) and 1B). We include this factor in the model by multiplying the rate of attachment, $k_{a}^{0}$, by the Boltzmann factor corresponding to the energy of the spring (as in [57]):

$$\begin{array}{c} k_{a}=k_{a}^{0}\exp[-\frac{1}{2}\frac{k}{k_{B}T}{\left(x_{i}^{\text{clu}}-x_{i}^{\text{nuc}}\right)}^{2}]. \end{array}$$ (1)

The positions of the cluster and nucleoid binding sites of the *i*-th PomZ dimer bound to the nucleoid and the PomXY cluster are given as $x_{i}^{\text{clu}}$ and $x_{i}^{\text{nuc}}$, respectively (see Fig 1B).

PomZ dimers bound to the PomXY cluster and the nucleoid are assumed to diffuse on both scaffolds (Fig 1A(4)). This assumption is motivated by two experimental observations. First, fluorescently tagged PomZ brightly stains the entire cluster in fluorescence micrographs [5]. Second, in a mutant with PomZ dimers that cannot bind to DNA, PomZ is homogeneously distributed inside the cell, which suggests that PomZ dimers only bind to the PomXY cluster when they are nucleoid-bound [5]. Based on these two experimental findings it seems reasonable that PomZ dimers are also mobile on the PomXY cluster as otherwise the concentration of PomZ would be rather concentrated at the cluster edges. PomZ can diffuse on the PomXY cluster and nucleoid with different diffusivities: We assume that the hopping rates are $\epsilon_{\text{hop},\;\text{nuc}}^{0}=D_{\text{nuc}}/a^{2}$ and $\epsilon_{\text{hop},\;\text{clu}}^{0}=D_{\text{clu}}/a^{2}$, with the lattice spacing *a*, respectively, being weighted by a Boltzmann factor that accounts for the energy change of the spring due to the movement:

$$\begin{array}{c} \epsilon_{\text{hop}}=\epsilon_{\text{hop}}^{0}\exp[-\frac{1}{4}\frac{k}{k_{B}T}({\left(x_{i}^{\text{clu},\;\text{to}}-x_{i}^{\text{nuc},\;\text{to}}\right)}^{2}-{\left(x_{i}^{\text{clu},\;\text{from}}-x_{i}^{\text{nuc},\;\text{from}}\right)}^{2})]. \end{array}$$ (2)

Here, $x_{i}^{\text{clu},\;\text{from}},x_{i}^{\text{nuc},\;\text{from}}$ and $x_{i}^{\text{clu},\;\text{to}},x_{i}^{\text{nuc},\;\text{to}}$ signify the position of the binding sites of the *i*-th PomZ dimer to the cluster and nucleoid before and after hopping, respectively. The additional factor of 1/2 in the exponent is chosen such that detailed balance holds for the hopping events and the rates for hopping to a neighboring site and hopping back are the inverse of each other (see [57]). A PomZ dimer is most likely to move in the direction that relaxes the spring (cf. exponential factor in Eq 2). We chose reflecting boundary conditions for diffusion of PomZ on both the nucleoid and the PomXY cluster.

Next, we discuss the transition from the nucleoid-bound state of PomZ to the cytosolic state. In the experiments, PomX, PomY and DNA stimulate the ATPase activity of PomZ, which leads to a conformational change and finally to detachment of two ADP-bound PomZ monomers from the nucleoid [5]. In our model, we combine the processes of nucleotide hydrolysis and detachment into one rate by assuming that nucleoid- and cluster-bound PomZ dimers are released into the cytosol with hydrolysis rate *k*_*h*_ (Fig 1A(5)). The ADP-bound PomZ monomers must then exchange ADP for ATP and dimerize before they can rebind to the nucleoid. This leads to a delay between detachment from and reattachment to the nucleoid (Fig 1A(6)). Because of this delay and rapid diffusion of PomZ in the cytosol [5] we assume that upon detachment of PomZ from the nucleoid, it can rebind to any lattice site of the nucleoid with the same probability. The total number of PomZ dimers is assumed to be constant and is denoted by *N*_total_.

So far we have described the stochastic dynamics of the PomZ dimers. The interactions of PomZ dimers with the PomXY cluster result in forces being exerted on the cluster, which cause it to move. The observable of interest is the cluster position, *x*_*c*_, over time. We approximate the cluster dynamics as overdamped, such that the equation of motion for *x*_*c*_ is given by the following force balance equation

$$\begin{array}{c} \gamma_{c}\partial_{t}x_{c}=-k\sum_{i=1}^{N}\left(x_{i}^{\text{clu}}-x_{i}^{\text{nuc}}\right), \end{array}$$ (3)

with *γ*_*c*_ being the friction coefficient of the PomXY cluster in the cytosol and *N* the total number of cluster-bound PomZ dimers. Experiments show that the Pom cluster displays very little motion in *M. xanthus* cells that lack PomZ, whereas its mobility is increased if PomZ is present [5]. Based on this observation, we disregard movements of the cluster due to thermal noise and focus on the stochasticity in the interactions of PomZ dimers with the PomXY cluster, which in turn lead to stochastic forces acting on the cluster. Therefore, we do not include a Langevin noise term in Eq 3.

### *In silico* parameter analysis

Our simulations show that the model indeed yields a robust mechanism for stochastic midnucleoid positioning of the PomXY cluster. The underlying mechanism for midnucleoid localization is based on the flux of PomZ on the nucleoid, which can be described as follows. If the PomXY cluster is located to the left of midnucleoid, the average flux of PomZ dimers into the cluster from the right is larger than that from the left [36] (red arrows in Fig 1A). If particles that attach to the cluster typically exert a net force in the direction from which they reached the cluster, the flux imbalance leads to a net force towards the right. For a cluster that overshoots midnucleoid or is already positioned to the right of midnucleoid, the asymmetry in the fluxes is reversed and the cluster moves back towards midnucleoid. Overall, this leads to a self-regulating process that positions the PomXY cluster at midnucleoid.

**Fig 2:**
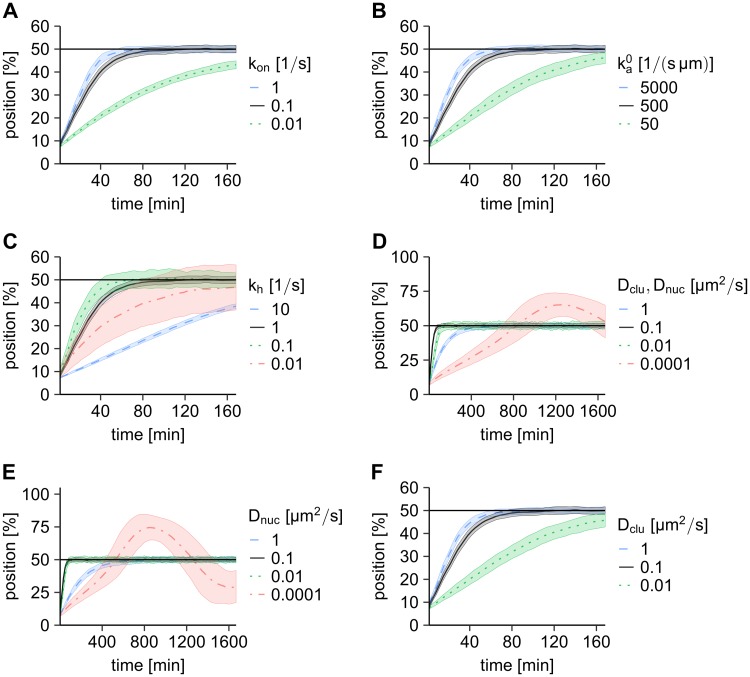
Exploring the parameter space. (A-F) Stochastic simulations show different qualitative behavior of the PomXY cluster trajectories when the model parameters are altered. We performed stochastic simulations using the parameter set given in S1 Table, with one of the parameters varied as indicated. In D, the diffusion constants of PomZ on the nucleoid and on the PomXY cluster are set to the same value. The result for the parameter set given in S1 Table is always shown in black for comparison purposes. The average cluster trajectories are shown as unbroken or dashed lines and the shaded regions indicate the region of ± one standard deviation. In the simulations, the initial position of the PomXY cluster is chosen such that the left edge of the cluster coincides with the left edge of the nucleoid (for more details see Materials and methods section). For the calculation of the mean and standard deviations the cluster positions are grouped into time intervals of 3.33 min. For each parameter set we simulated at least 100 trajectories.

The stochastic simulations show midnucleoid positioning of the PomXY cluster (Fig 2, data shown in black) with physiologically relevant parameters (S1 Table, for the discussion of the parameters see S1 Text). To identify the parameter range that leads to midcell localization and investigate the role of each parameter on the cluster dynamics, we performed broad parameter sweeps. We varied the attachment rate of PomZ dimers to the nucleoid, *k*_on_, the binding rate of nucleoid-bound PomZ dimers to the PomXY cluster, $k_{a}^{0}$, the ATP hydrolysis rate of PomZ dimers, *k*_*h*_, and the mobility of PomZ dimers on the nucleoid, *D*_nuc_, and on the PomXY cluster, *D*_clu_, over a broad range (Fig 2). We never restricted the PomZ dimer density on the nucleoid and bound to the cluster, as we can assume that these densities are low in the wild type situation with a total number of PomZ dimers of *N*_total_ ≈ 100 [5].

The parameter sweeps show that increasing the attachment rate to the nucleoid, *k*_on_, or the binding rate to the PomXY cluster, $k_{a}^{0}$, decreases the time the cluster needs to reach midnucleoid (Fig 2A and 2B). In both cases, the trajectories become independent of the particular parameter when its value exceeds a certain threshold. We conclude that increasing the rate of attachment of PomZ to the nucleoid or the binding of PomZ to the PomXY cluster speeds up the positioning process until an optimum is reached.

Next, we consider the effects of varying the rate of ATP hydrolysis by PomZ dimers associated with the PomXY cluster, which is important to maintain the cyclic flux of PomZ dimers between the cytosolic and nucleoid-bound state. This rate also sets the time scale for the interaction of PomZ dimers with the PomXY cluster. The simulations show that decreasing the hydrolysis rate (*k*_*h*_ = 0.01 s^−1^) reduces the velocity of the average cluster trajectory towards midnucleoid (Fig 2C). Qualitatively, large hydrolysis rates (*k*_*h*_ = 10 s^−1^) have essentially the same effect (Fig 2C). Hence, there is a hydrolysis rate *k*_*h*_ that minimizes the time the cluster needs to reach midnucleoid. Although the average cluster trajectory behaves similarly for large and small hydrolysis rates, we observe that the variance of the cluster distribution over time decreases with increasing hydrolysis rate (Fig 2C).

Apart from the ATP hydrolysis rate, we expect the diffusivity of PomZ on the nucleoid to be a crucial parameter for cluster movement, because it determines the time needed for PomZ dimers to explore the nucleoid to the left or right of the cluster. Interestingly, when we reduce the diffusivity of PomZ on the nucleoid in the simulations, the clusters begin to oscillate around the midnucleoid position (Fig 2D, 2E and S1 Fig). Finally, we also decreased the diffusion constant of PomZ dimers on the PomXY cluster, while keeping the diffusion constant on the nucleoid fixed. In this case, the clusters take longer to reach midcell (Fig 2F).

In addition to the parameter sweeps shown in Fig 2, we also considered the PomXY cluster trajectories when the spring stiffness *k* and the total PomZ dimer number *N*_total_ are varied. The cluster trajectories do not change significantly when the spring stiffness is altered over one order of magnitude, and an increase in the particle number increases the velocity of cluster movement towards midcell (S2 Fig). Furthermore, we verified that the results do not change qualitatively in an extension of the stochastic model that includes a small detachment rate of nucleoid-, but not cluster-bound PomZ dimers. For very large detachment rates that are above the expected values from experiments midcell localization of the cluster breaks down (see S1 Text and S3 Fig).

To summarize, we observed that there exists an ATP hydrolysis rate that minimizes the time taken to reach midnucleoid. The diffusion constant of PomZ on the nucleoid determines whether the PomXY cluster moves towards or oscillates around midnucleoid. Moreover, the clusters move faster towards midcell if PomZ dimers diffuse faster on the PomXY cluster. In the following, we provide first an analytic approach that explains our observations regarding the cluster dynamics when the ATP hydrolysis rate and the diffusion constant of PomZ on the cluster is varied. We then consider the oscillatory cluster dynamics and give an estimate for the onset of oscillations.

### A deterministic approximation for the average cluster trajectory

Our goal is to understand what generates the force behind midcell positioning in our model. We expect that the cyclic flow of PomZ dimers is at the root of this force: PomZ dimers attach to the nucleoid in their active state (as ATP-bound PomZ dimers), diffuse on the nucleoid and are released into the cytosol in their inactive state (ADP-bound PomZ monomers) after encountering the PomXY cluster. We describe how the cyclic flow can lead to a net force in the following.

We assume that the PomZ dynamics is fast compared to the PomXY cluster dynamics (adiabatic assumption), and approximate the system by a stationary model, i.e. a system with a fixed cluster position. As we neglect exclusion effects on the nucleoid, PomZ dimers can only interact with each other via the PomXY cluster. However, when the cluster is stationary, no interaction between the cluster-bound PomZ dimers is possible, and thus the movements of different PomZ proteins are not correlated. Therefore, we can consider the interactions of PomZ dimers with the PomXY cluster as independent, which yields the following deterministic approximation for the total net force, *F*, acting on a cluster at position *x*_*c*_

$$\begin{array}{c} F\left(x_{c}\right)=\left(N_{R}\left(x_{c}\right)-N_{L}\left(x_{c}\right)\right)f, \end{array}$$ (4)

with *f* being the time-averaged force exerted by a single PomZ dimer that attaches to the nucleoid on the right side of the cluster. For symmetry reasons, a PomZ dimer coming from the left then exerts a time-averaged force −*f*. *N*_*R*_ and *N*_*L*_ denote the numbers of PomZ dimers that are bound to the cluster and had originally attached to the nucleoid to the right and left of the cluster, respectively. These two numbers increase with the diffusive flux of nucleoid-bound PomZ dimers reaching the cluster region from the right and left side, *j*_*R*/*L*_, respectively, and decrease with the ATP hydrolysis rate, *k*_*h*_, as long as the attachment rate to the PomXY cluster is non-zero. Hence, we obtain

$$\begin{array}{c} N_{R/L}\left(x_{c}\right)=\frac{j_{R/L}\left(x_{c}\right)}{k_{h}} \end{array}$$ (5)

in the steady-state. Inserting this into Eq 4, yields

$$\begin{array}{c} F\left(x_{c}\right)=\frac{j_{R}\left(x_{c}\right)-j_{L}\left(x_{c}\right)}{k_{h}}f=\frac{f}{k_{h}}j_{\text{diff}}\left(x_{c}\right)\equiv Cj_{\text{diff}}\left(x_{c}\right). \end{array}$$ (6)

We conclude that the net force is proportional to the flux difference of PomZ dimers at the cluster, *j*_diff_, and the proportionality constant is given by *C* = *f*/*k*_*h*_. Importantly, simulation results with a fixed cluster position confirm the observation that the total force exerted on the cluster is proportional to the PomZ flux difference (S4 Fig).

Next, we investigate how the net force exerted on the PomXY cluster results in movement of the cluster. Notably, the PomZ dimers interacting with the cluster not only produce a net force on the cluster, they also reduce the mobility of the cluster by tethering it to the nucleoid. We assume that these two processes can be considered independently. We simulated the steady-state velocity with which a cluster moves when a fixed number of PomZ dimers are bound to it and an external force is applied to the cluster (see Materials and methods section for details). We found that this velocity varies linearly with the force (S5 Fig), which suggests that the force exerted on the cluster is balanced by a frictional force with effective friction coefficient *γ*(*x*_*c*_): *F*(*x*_*c*_) = *γ*(*x*_*c*_)*v*(*x*_*c*_).

With Eq 6 we obtain the central equation of our analysis

$$\begin{array}{c} v\left(x_{c}\right)=\frac{F(x_{c})}{\gamma (x_{c})}=\frac{Cj_{\text{diff}}\left(x_{c}\right)}{\gamma (x_{c})}, \end{array}$$ (7)

which relates the average velocity of the cluster to the flux difference of PomZ dimers into the cluster, the proportionality constant *C* and the effective friction coefficient *γ*(*x*_*c*_) of the cluster. To obtain the average cluster trajectory, we need to integrate Eq 7 over time. In the following we derive analytical expressions for the flux difference into the cluster and the effective friction coefficient of the PomXY cluster. The constant *C* we determine from simulations. Since *C* does not change with the cluster position, *x*_*c*_, the dependence of the velocity on *x*_*c*_ is given by an analytical expression, which can be integrated (numerically).

#### Analytical expression for the PomZ flux difference

We start by deriving an analytical expression for the difference in PomZ flux into the cluster, *j*_diff_, in the adiabatic limit. To do so, we introduce a reaction-diffusion (RD) model that simplifies the analysis compared to our stochastic model.

In this model, the nucleoid is reduced to a one-dimensional line of length *L* and the PomXY cluster is a finite interval on this line, *I*_*c*_ = [*x*_*c*_ − *L*_*c*_/2, *x*_*c*_ + *L*_*c*_/2]. Let *c*(*x*, *t*) denote the concentration of PomZ dimers bound to the nucleoid only, *c*_*b*_(*x*, *t*) the concentration of PomZ dimers bound to the nucleoid and cluster, and *N*_cyto_ the number of PomZ dimers in the cytosol. The nucleoid and cluster are assumed to have reflecting boundary conditions for the nucleoid-bound and cluster-bound PomZ dimers, respectively. In accordance with the stochastic model, PomZ dimers attach to the nucleoid left and right of the cluster with rate *k*_on_ and diffuse on the nucleoid with diffusion constant *D*_nuc_. In the RD model we simplify the interactions of PomZ dimers with the cluster: nucleoid-bound PomZ in the cluster region, *I*_*c*_, can bind to the cluster with a rate $k_{a}^{\text{total}}$, neglecting the elasticity of the PomZ dimers and the chromosome included in our stochastic model (for details see S2 Text):

$$\begin{array}{c} k_{a}^{\text{total}}=\{\begin{array}{cc} k_{a}^{0}\sqrt{\frac{2\pi k_{B}T}{k}}, & x\in I_{c},\\ 0, & \text{otherwise}. \end{array} \end{array}$$ (8)

We assume that PomZ dimers bound to the cluster and the nucleoid diffuse with a diffusion constant *D*_*b*_ = 0.5 *D*_nuc_ (see S2 Text). Finally, PomZ dimers bound to the cluster and nucleoid can hydrolyze ATP and subsequently detach into the cytosol with rate *k*_*h*_. With these model assumptions we obtain the following reaction-diffusion equations to describe the PomZ dynamics, respecting particle number conservation:

$$\begin{array}{ccc} \partial_{t}c\left(x,t\right)=D_{\text{nuc}}\partial_{x}^{2}c\left(x,t\right)+\frac{k_{\text{on}}N_{\text{cyto}}\left(t\right)}{L}, & & \left(x\notin I_{c}\right) \end{array}$$ (9)

$$\begin{array}{ccc} \partial_{t}c\left(x,t\right)=D_{\text{nuc}}\partial_{x}^{2}c\left(x,t\right)-k_{a}^{\text{total}}c\left(x,t\right), & & \left(x\in I_{c}\right) \end{array}$$ (10)

$$\begin{array}{ccc} \partial_{t}c_{b}\left(x,t\right)=D_{b}\partial_{x}^{2}c_{b}\left(x,t\right)+k_{a}^{\text{total}}c\left(x,t\right)-k_{h}c_{b}\left(x,t\right), & & \left(x\in I_{c}\right) \end{array}$$ (11)

$$\begin{array}{c} \partial_{t}N_{\text{cyto}}\left(t\right)=k_{h}\int_{x_{c}-L_{c}/2}^{x_{c}+L_{c}/2}dx\;c_{b}\left(x,t\right)-k_{\text{on}}\frac{L-L_{c}}{L}N_{\text{cyto}}\left(t\right), \end{array}$$ (12)

with the following no-flux boundary conditions at the nucleoid and cluster edges:

$$\begin{array}{c} \partial_{x}{c\left(x,t\right)|}_{x=0}=0=\partial_{x}{c\left(x,t\right)|}_{x=L}, \end{array}$$ (13)

$$\begin{array}{c} \partial_{x}c_{b}{\left(x,t\right)|}_{x=x_{c}-L_{c}/2}=0=\partial_{x}c_{b}{\left(x,t\right)|}_{x=x_{c}+L_{c}/2}. \end{array}$$ (14)

**Fig 3:**
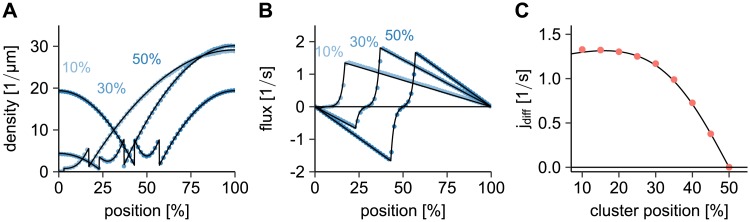
Comparison of the RD with the stochastic model. (A, B) Density and flux of PomZ dimers along the nucleoid for PomXY clusters at 10%, 30% and 50% nucleoid length. For the PomZ density we use the nucleoid binding site as the PomZ dimer position if PomZ is nucleoid-bound only and the cluster binding site if PomZ is bound to the cluster. Regarding the flux, only PomZ dimers bound to the nucleoid, but not the cluster, are considered. The analytical result obtained from the RD equations is shown in black and the results from the stochastic simulations in blue. (C) PomZ flux difference into the cluster as a function of the cluster position. The black line indicates the result from the RD equation, the red points are results from the stochastic simulations. For the data shown in this Figure we simulated 100 cluster trajectories with parameters as in S1 Table. See the Materials and methods section for more details.

The stationary state of this RD model yields a PomZ density and flux on the nucleoid that agrees well with the stochastic simulation results for the parameter values given in S1 Table (Fig 3). For details on the stationary solution of the above equations we refer to S2 Text. We observe that the density and flux profiles are asymmetric for off-center clusters, but become increasingly symmetric as the cluster approaches midnucleoid (Fig 3A and 3B). This leads to a diffusive flux difference into the cluster that decreases towards midnucleoid (Fig 3C). Note that the flux difference decreases slightly for clusters close to the nucleoid pole, which is due to total particle number conservation in the system.

#### Force exerted by a single PomZ dimer

Next, we investigate the force exerted by a single PomZ dimer on the PomXY cluster. How can the interaction of a PomZ dimer with the PomXY cluster lead to a net force? PomZ dimers can exert a net force by attaching to the PomXY cluster in a stretched configuration (as in the DNA-relay mechanism, [39]): a particle to the left / right of the cluster can bind to the cluster from a position beyond either end (Fig 1B). In addition, PomZ dimers can impart a force to the cluster, when they reach the edge of the cluster, because PomZ dimers interacting with the PomXY cluster can diffuse on both the nucleoid and cluster, but the cluster binding site is restricted in its movement due to the boundaries (Fig 1B).

To investigate the force generated by a single PomZ dimer, we performed stochastic simulations with a stationary PomXY cluster and only one PomZ dimer in the system (for details see Materials and methods). We only consider particles that attach to the nucleoid at the right side of the cluster. The constant *C* in Eq 7 is given by the time-averaged one-particle force *f* divided by the ATP hydrolysis rate *k*_*h*_ (Eq 6). In our simulation, we determined the ensemble average of the time-averaged forces using the interaction times as weights, which results in a constant *C* = 0.0059 pN s for the parameters as in S1 Table (see Materials and methods for details). Note that this value for *C* matches with the proportionality constant between the total force exerted on the PomXY cluster and the PomZ flux difference for a stationary cluster, *C* = 0.0059 pN s (S4 Fig). Importantly, the constant *C* and hence the ensemble average of the time-averaged force of PomZ dimers attaching from the right is greater than zero. Thus a particle coming from the right exerts indeed, on average, a force directed to the right and vice versa (S6 Fig, parameters as in S1 Table). We also determined the average distance, Δ*x*_0_, between the nucleoid and cluster binding site when the particle attaches to the cluster in our simulations. The value we obtained, Δ*x*_0_ ≈ 0.0011 μm, is very small and even less than one lattice spacing, *a* = 0.01 μm. Based on this small deflection we can conclude that the main contribution to the force exerted on the cluster is not the force due to the initial deflection of the PomZ dimer spring, but the force exerted at the cluster’s edges while the PomZ dimer is bound to the cluster (for details see S3 Text).

We now have approximations for the flux difference into the cluster (results from Eqs 9–14) and the proportionality constant between the force and the flux difference (Eq 6). The only parameter yet to be estimated is the effective friction coefficient of the PomXY cluster, which we consider next.

#### Effective friction coefficient of the PomXY cluster

We derived an analytical expression for the effective friction coefficient by assuming that the cluster and the nucleoid boundaries can be disregarded (see S4 Text). We find that the effective friction coefficient of the cluster is given by the cytosolic friction coefficient plus a term that increases linearly with the number, *N*, of PomZ dimers bound to the cluster:

$$\begin{array}{c} \gamma \left(x_{c}\right)=\gamma_{c}+\frac{k_{B}TN\left(x_{c}\right)}{D_{\text{clu}}+D_{\text{nuc}}}. \end{array}$$ (15)

The increase with *N* is due to the fact that the more PomZ dimers tether the cluster to the nucleoid, the more restricted it is in its movement. Note that the friction coefficient depends on the cluster position *x*_*c*_, because the number of PomZ dimers attached to the cluster changes with *x*_*c*_. Furthermore, we find that the larger the diffusion constant of PomZ dimers on the nucleoid and the cluster, the smaller the additional contribution to the friction coefficient *γ*_*c*_. This can be attributed to the fact that the PomZ dimers restrict the cluster’s movement less strongly the more mobile they are. Our analytical result agrees with the simulation results for an infinitely extended cluster and nucleoid and a constant number *N* of PomZ dimers bound to the cluster (S7 Fig, for details see Materials and methods section). In general, an approximation for the number of cluster-bound PomZ dimers can be obtained from the stationary solution of the RD model (see S2 Text). With the friction coefficient of the PomXY cluster we now have estimates for all factors that contribute to the velocity of the PomXY cluster (Eq 7) and hence determine the average cluster trajectory.

#### Semi-analytical approach explains observed simulation results

**Fig 4:**
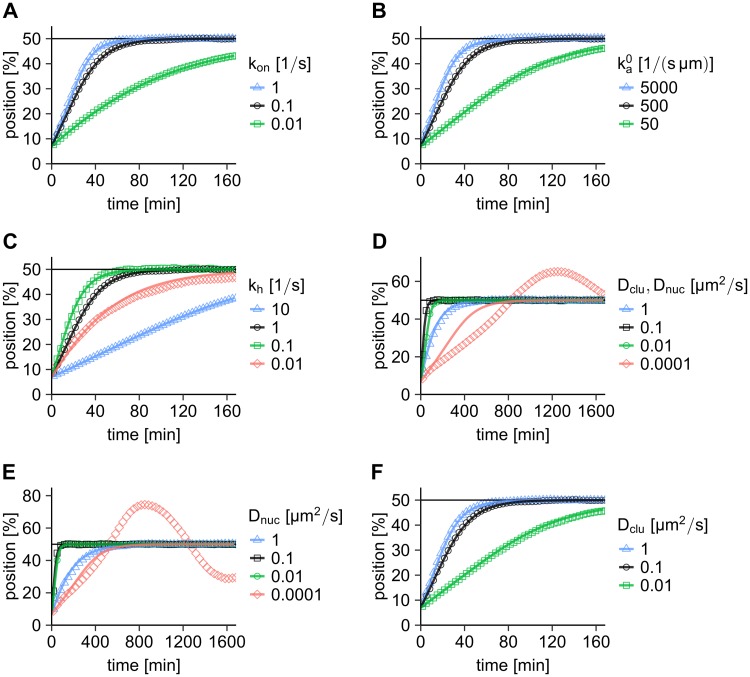
Comparison of the average cluster trajectory from simulations with our semi-analytical approximation. (A-F) The cluster trajectories obtained from integrating the equation of motion of the PomXY cluster, Eq 7, (solid lines) agree with the simulation results for most parameters (points of different shape, same data as shown in Fig 2). In the semi-analytical approximation we use the theoretical values for the flux difference and the friction coefficient together with the simulated value for *C*. For the parameters for which the cluster overshoots midcell (small *D*_nuc_), our semi-analytical theory does not match the simulation results. This is expected, because we make the assumption that the PomZ dimer dynamics is faster than the cluster movement (adiabatic assumption). If not explicitly given in the Figure, the parameters are as in S1 Table.

Using the analytical values for the PomZ flux difference at the cluster boundaries, the effective friction coefficient of the PomXY cluster, and the simulated values for the force exerted by a single particle on the PomXY cluster, we can obtain an estimate for the average cluster trajectory. For most of the parameters, the simulated average cluster trajectory and the approximation from our semi-analytical approach are in good agreement (Fig 4A–4F, S8 Fig). There are some parameter combinations, where our approximation lies above the simulation results. This is probably due to the fact that the dynamics of the PomXY cluster and the PomZ dimers cannot be separated in these cases. For example for low ATP hydrolysis rates, the net force exerted by PomZ on the cluster is smaller than captured in our approximation, because PomZ dimers are bound to the cluster for a long time such that a forward movement of the cluster can lead to an accumulation of PomZ at the trailing edge of the cluster, which decreases the velocity towards midcell. When the PomZ dynamics is slow compared to the cluster dynamics, as is the case for small *D*_nuc_, the adiabatic assumption breaks down, and our semi-analytical approach fails to reproduce the simulated cluster trajectories (Fig 4D and 4E).

The semi-analytical approach enables us to gain further mechanistic insights into the regulation of the cluster dynamics by PomZ dimers. The good agreement between the simulated cluster trajectories and our estimates from this approach, for most of the parameters (Fig 4), shows that the average cluster dynamics can be described solely by the PomZ flux difference into the cluster, the force a single PomZ dimer exerts on the cluster and the effective friction coefficient. Now we use this approach to get further insights into the cluster dynamics when the ATP hydrolysis rate and the diffusion constant of PomZ on the PomXY cluster are varied, two parameters that showed interesting behavior in the parameter sweeps (Fig 2C and 2F).

**Fig 5:**
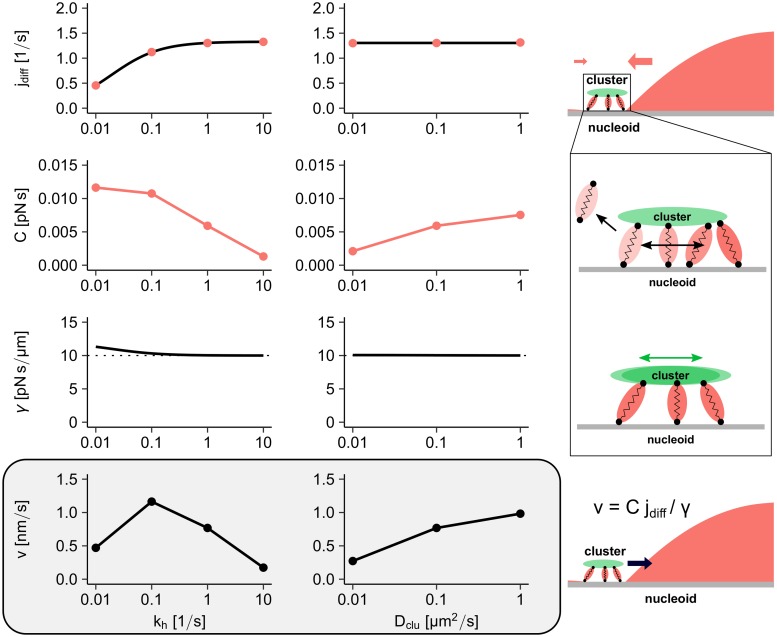
Force generation in the flux-based model. The average velocity of the cluster is approximated by the difference in flux of PomZ dimers into the cluster region from either side, *j*_diff_, the constant *C*, which describes the force exerted by a single PomZ dimer on the PomXY cluster, and the effective friction coefficient of the cluster, *γ*. Here, the impact of varying the hydrolysis rate *k*_*h*_ (first column) or the diffusion constant of PomZ dimers on the PomXY cluster, *D*_clu_ (second column) is shown. The first row shows the PomZ flux difference at the cluster when the cluster is at 20% nucleoid length. The result from the RD equations (black line) matches the stochastic simulation results (red points). The second row shows the proportionality constant *C* determined from one-particle simulations (more than 40000 PomZ dimer-cluster interactions are simulated). The points are connected by lines to guide the eye. The third row shows the analytical curves for the effective friction coefficient of the PomXY cluster at 20% nucleoid length obtained from Eq 15. An increase in the number of PomZ dimers bound to the cluster (e.g. for low *k*_*h*_ values) leads to effective friction coefficients larger than the cytosolic friction coefficient (dotted horizontal line). Finally, the average velocity of the cluster can be calculated based on the flux difference, the constant *C* and the friction coefficient using Eq 7. The velocity obtained using the theoretical values for both the flux difference and the friction coefficient, and the simulated values for *C*, is shown in the last row (grey box). The points are connected by lines to guide the eye. If not explicitly given in the Figure, the parameters are as in S1 Table. See the Materials and methods section for more details.

Fig 5 gives an overview of the different contributions to the cluster’s velocity when the ATP hydrolysis rate, *k*_*h*_, or the diffusion constant of PomZ on the PomXY cluster, *D*_clu_, is varied (for further parameters see S9 and S10 Figs). The flux difference of PomZ dimers into the cluster “measures” the position of the cluster on the nucleoid (first row). Cluster-bound PomZ dimers exert a net force upon encountering the cluster’s edge and they increase the friction of the PomXY cluster by tethering it to the nucleoid (second and third row). Taken together, a difference in the PomZ fluxes into the cluster and local force generation by PomZ dimers at the PomXY cluster boundaries impart a velocity to the cluster that leads to a net movement towards midnucleoid (fourth row).

Increasing the ATP hydrolysis rate increases the flux difference, because it determines the rate of PomZ dimer release from the nucleoid, and hence is important for the flux of PomZ dimers through the system (Fig 5). To understand the dependence of *C* = *f*/*k*_*h*_ on *k*_*h*_, we first consider the dependence of the time-averaged force *f* on *k*_*h*_. The time-averaged force *f* increases with *k*_*h*_ because *k*_*h*_ sets the time scale of interaction with the cluster: particles exert on average a larger force initially, so that shorter averaging (larger *k*_*h*_) leads to a larger time-averaged force (see S11 Fig). Nevertheless, this increase is less than linear in *k*_*h*_, such that *C* = *f*/*k*_*h*_ decreases with *k*_*h*_ (Fig 5). The effective friction coefficient also decreases with increasing *k*_*h*_, because the number of PomZ dimers bound to the PomXY cluster decreases. The increasing flux difference, the decreasing constant *C*, and a decrease in friction together result in a maximal velocity, *v*, of the cluster for intermediate *k*_*h*_ values (Fig 5). This explains why there exists a hydrolysis rate for which the cluster trajectory reaches midnucleoid in a minimal time (Fig 2C). Furthermore, we observed that the variance in the cluster position decreases with increasing *k*_*h*_. Since an increase in the hydrolysis rate increases the flux of PomZ dimers through the system and decreases the interaction time of PomZ dimers with the PomXY cluster, we expect a less stochastic movement of the cluster for larger hydrolysis rates, as observed.

Furthermore, we considered the case where the PomZ diffusion constant on the PomXY cluster is reduced while keeping the diffusion constant on the nucleoid fixed. Since diffusion on the PomXY cluster only affects the PomZ dynamics locally at the cluster, changing this rate does not alter the flux difference of PomZ into the cluster (Fig 5), but it does alter the magnitude of force generation at the cluster. We find that the time-averaged one-particle force decreases with decreasing diffusion constant (Fig 5), which explains the increase in the time required for a cluster to reach midnucleoid for small diffusion constants (Fig 2F). Why the force decreases when the diffusion constant on the PomXY cluster is reduced can be understood intuitively as follows: Our findings indicate that the main contribution to the net force generated by the PomZ dimers is the force they exert when they encounter the cluster’s edge. When the diffusion constant of PomZ on the PomXY cluster, *D*_clu_, is zero, the nucleoid binding site of a cluster-bound PomZ dimer equilibrates and fluctuates around this equilibrium position without producing a net force. Hence, in this case only attachment of PomZ dimers to the cluster in a stretched state results in a net force.

To summarize, with our semi-analytical approach we can get new mechanistic insights into the cluster dynamics. In this approach we separate the global asymmetry, i.e. a cluster located at an off-center position, which results in different diffusive PomZ fluxes into the cluster, from the forces locally exerted on the cluster. In particular, we can identify the different contributions to the velocity of the cluster and thereby understand why there is an ATP hydrolysis rate that results in a minimal time the clusters need to reach midcell and why diffusion of PomZ dimers on the PomXY cluster matters in our model.

### Oscillatory behavior vs. midnucleoid localization of the cluster

We observe a marked discrepancy between the simulated average cluster trajectory and our approximation when the diffusion constant of PomZ on the nucleoid is reduced and the cluster oscillates around midnucleoid (Fig 4D and 4E). Deviations from our theoretical predictions are to be expected in this situation, because we make an adiabatic assumption in our semi-analytical approach, i.e. we assume that the PomZ dimer dynamics on the nucleoid is fast compared to the cluster movement. This assumption no longer holds when PomZ dimers diffuse slowly on the nucleoid. In this case, the distribution of PomZ density along the nucleoid determined from simulations with a dynamic cluster deviates drastically from its steady-state distribution (S12 Fig). If the cluster initially lies to the left of midnucleoid and approaches midnucleoid from that side, our theory predicts a symmetric PomZ density, whereas the simulations show a higher density in front of the cluster. The flux difference also deviates from the stationary case: it increases as the cluster moves towards midnucleoid instead of vanishing at midnucleoid (S12 Fig). Both the asymmetric density and the non-zero flux difference at midnucleoid are in accordance with the observed oscillatory behavior.

**Fig 6:**
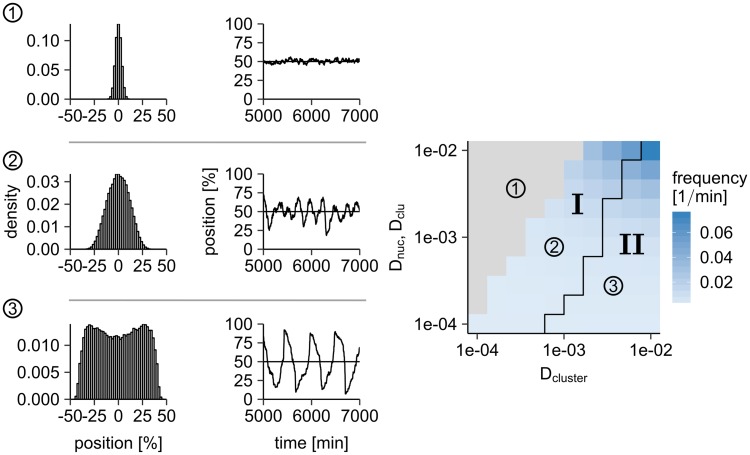
Oscillatory cluster movement occurs if PomZ dynamics is slower than PomXY cluster dynamics. We varied the diffusion constant of the PomXY cluster, *D*_cluster_ = *k*_*B*_*T*/*γ*_*c*_, and the diffusion constant of PomZ on the nucleoid and PomXY cluster (*D*_clu_ and *D*_nuc_, are set to the same value). The other parameters are as in S1 Table. The clusters localize at midnucleoid for high PomZ diffusion constants and low diffusion constants of the cluster *D*_cluster_ (grey region). If the diffusion constant of PomZ is decreased from 0.01 μm^2^/s, the clusters begin to oscillate, because the time scales of the PomZ dimer dynamics and the cluster dynamics become comparable (region I). The average frequency of oscillation is shown in blue (100 runs per parameter set are considered). In this parameter regime, the distribution of cluster positions is peaked around midnucleoid (see histograms on the left for the parameters marked in the phase diagram). For even lower PomZ diffusion constants and relatively large diffusion constants of the PomXY cluster (region II) the cluster positions are bimodally distributed. In the simulations, the clusters begin at midnucleoid and are recorded for 10000 min. The black line in the frequency plot indicates a threshold. Below the curve the cluster distribution is bimodal, above it the distribution has only one peak. For details of the data analysis see the Materials and methods section.

The switch between cluster localization at midnucleoid and oscillatory movement around midnucleoid is regulated by the relative time scales of PomZ dynamics and cluster dynamics: If the PomXY cluster is moving slowly or the PomZ dimers move fast, the latter have time to adjust to a change in the cluster position. On the other hand, if the cluster moves fast or the PomZ dimers move slowly, the PomZ dimer distribution deviates from the stationary case. The delay between the movement of the cluster and the build-up of the PomZ gradient, which in turn biases the movement of the cluster, leads to oscillations: the longer the delay, the larger the amplitudes of the oscillations. To investigate the oscillatory case further, we performed additional simulations in which the diffusion constant of the PomZ dimers and that of the PomXY cluster in the cytosol, which is inversely proportional to the friction coefficient, *γ*_*c*_, according to the Stokes-Einstein relation, were varied. As expected, we find oscillatory behavior of the clusters for low diffusion constants of PomZ on the nucleoid (Fig 6). In the oscillatory regime we find both bimodal and monomodal cluster position distributions (Fig 6).

As mentioned above, the onset of oscillations depends on the time scales of PomZ gradient formation and cluster movement. In order to understand how the parameters change the behavior of the cluster trajectory, i.e. lead to oscillatory movement or midcell positioning, we assume that the cluster is located at midnucleoid and search for a stability condition that distinguishes the two behaviors. The diffusion time for a PomZ dimer to explore a nucleoid of size *L* is given by

$$\begin{array}{c} t_{\text{PomZ}}=\frac{L^{2}}{D_{\text{nuc}}}. \end{array}$$ (16)

In theory, the velocity of a cluster that starts from midnucleoid should be zero, because there should be no difference between the fluxes of PomZ dimers from both sides. However, due to stochastic effects, more particles may attach to the cluster from the right than from the left side, which will displace the cluster to the right. For our time scale argument, we consider an extreme case: we assume that PomZ dimers only arrive from one side, which we choose to be the right side without loss of generality. The time required for a cluster to move the length of the nucleoid is then given by

$$\begin{array}{c} t_{\text{cluster}}=\frac{L}{v}\approx \frac{L\gamma (0.5L)}{Cj_{\text{right}}\left(0.5L\right)}, \end{array}$$ (17)

with *j*_right_ being the flux of PomZ dimers into the cluster from the right. Here, we approximate the velocity of the cluster by its effective description, Eq 7, using *x*_*c*_ = 0.5*L*, and replace the flux difference with the flux from the right only. According to Eq 17, the condition for stable positioning of the cluster at midnucleoid

$$\begin{array}{c} t_{\text{PomZ}}\ll t_{\text{cluster}} \end{array}$$ (18)

yields

$$\begin{array}{c} \frac{D_{\text{nuc}}}{L}\gg \frac{Cj_{\text{right}}\left(0.5L\right)}{\gamma (0.5L)}=\frac{Cj_{\text{right}}\left(0.5L\right)}{\gamma_{c}+\left(k_{B}TN\left(0.5L\right)\right)/\left(D_{\text{clu}}+D_{\text{nuc}}\right)}. \end{array}$$ (19)

For the parameter sweeps considered before (Fig 2 and S2 Fig), we find *t*_cluster_ ≫ *t*_PomZ_ for all cases except for small diffusion constants of PomZ on the nucleoid.

With our time-scale argument, Eq 19, we can make further predictions as to which parameters should result in oscillations. First, we consider a change in the total particle number, *N*_total_. Both *j*_right_ as well as the number of cluster-bound proteins, *N*, are proportional to *N*_total_, and *C* does not depend on *N*_total_. Therefore, the right-hand side of Eq 19 is proportional to *N*_total_ for small values of *N*_total_ and converges to a constant for large values. From this we expect that oscillatory behavior may occur for large particle numbers. Simulations with 500 PomZ dimers and a smaller diffusion constant of PomZ on the nucleoid and the PomXY cluster compared to the parameters in S1 Table (*D*_nuc_ = *D*_clu_ = 0.01 μm^2^/s) indeed show oscillatory behavior, whereas simulations with the same parameters, but 100 PomZ dimers show midnucleoid localization (S13 Fig). However, for very large PomZ dimer numbers we expect exclusion effects, which are not considered here, to have an impact that will also affect the cluster dynamics.

Second, we investigate the effects on the cluster dynamics when changing the nucleoid length *L*. Again, the constant *C*, which represents the force exerted by a single PomZ dimer on the PomXY cluster, does not depend on *L*. The number of cluster-bound proteins decreases with increasing *L*, because the relative size of the cluster *L*_*c*_/*L* decreases and the total PomZ dimer number in the system is constant. Hence, also the flux of PomZ in the system is reduced, which leads to a decrease of the flux *j*_right_ with increasing *L*. Bringing all terms in Eq 19 that depend on *L* to the right hand side yields a curve that first increases with *L*, then reaches a maximum and decreases again for large *L*. Hence, we expect no oscillations for small and large nucleoid lengths and oscillations might occur for intermediate lengths. Simulations with intermediate and large nucleoid lengths *L* indeed show this behavior (S13 Fig).

## Discussion

We analyzed how the cluster movement changes when the rates for the key biological processes are varied over a broad range. We found that there exists an optimal ATP hydrolysis rate of PomZ such that the time the cluster needs to move to midnucleoid is minimized. A parameter sweep of the diffusion constant of PomZ on the PomXY cluster shows that the mobility of PomZ dimers on the PomXY cluster is important for cluster movement towards midnucleoid. Qualitative changes in the cluster trajectories are observed when the diffusion constant of PomZ on the nucleoid is reduced: midnucleoid positioning of the cluster switches to oscillatory behavior of the cluster around midnucleoid. Hence, we conclude that positioning of the cluster in the flux-based model critically depends on the time scale for the cluster dynamics in comparison to the one for the PomZ dimer dynamics on the nucleoid. If the latter is slow compared to the cluster dynamics, the cluster will oscillate around midnucleoid. In contrast, fast PomZ dynamics on the nucleoid leads to midnucleoid localization of the cluster. In the latter case, we find that the average velocity of the PomXY cluster can be described by the PomZ flux difference into the cluster, which measures how far away the cluster is from midnucleoid, the force exerted by a single PomZ dimer on the cluster, and the effective friction coefficient of the cluster, which depends on the number of PomZ dimers bound to it (semi-analytical approach). This approach allows for further mechanistic insights into the cluster movement by PomZ dimer interactions. With it we can explain the dependence of the cluster dynamics on the model parameters as observed in our simulations.

The mechanism we propose for midcell localization of the Pom cluster in *M. xanthus* is based on a flux-balance argument, which was previously proposed for positioning by the Par system [36] and also for self-organized positioning of protein clusters that dynamically form on the nucleoid [52]. In the model by Ietswaart et al. [36] and the model we present here, the cargo is a fixed structure, whereas Murray and Sourjik [52] consider a reaction-diffusion model for a protein that can form dynamic clusters on the nucleoid, which are positioned by the same protein due to a flux-balance argument. Necessary conditions for flux-based positioning are that the ATPase diffuses on the nucleoid (faster than the cargo) and cycles between a nucleoid-bound and cytosolic state [5, 36, 52]. Furthermore, the typical length an ATPase diffuses on the nucleoid before it detaches into the cytosol (without a preceded interaction with the cargo) has to be sufficiently large compared to the nucleoid length to ensure positioning of a cargo at midcell (see S3 Fig, S1 Text) [52, 58].

How the forces are generated by the ParA-like ATPase to move the cargo (plasmid, partition complex or protein cluster) is still under debate. Lim et al. proposed that forces are generated due to the elasticity of the nucleoid [39], which we also assume here. Alternatively, a chemophoretic force has been suggested. Chemophoretic forces can explain the net movement of catalytic particles in the direction of an increasing concentration of a solute [58] and have also been applied to positioning of cargoes by the Par system [47, 53–55, 58]. To what extent a chemophoretic force and / or the elasticity of the nucleoid lead to the net force that moves the cargoes remains to be investigated.

One important experimental observation that differs between the Pom system and several Par systems is that PomZ dimers accumulate at the cluster. In our model, we make two important model assumptions that affect the density profile of PomZ at the cluster: First, we assume that cluster-bound PomZ dimers can only detach from the cluster via ATP hydrolysis, such that the dimers are captured at the cluster until they are released into the cytosol. Second, we assume that cluster-bound PomZ dimers can diffuse on both the cluster and the nucleoid. These assumptions have important implications on how forces are generated at the cluster in our model.

We find that the PomZ dimer springs not only exert forces when they attach to the cluster in a stretched configuration (as in the DNA-relay model, [39]), but instead forces can be generated every time a cluster-bound PomZ dimer encounters the cluster’s edge. Our simulations show that the latter contribution to the overall force of a single PomZ dimer is much more important than binding in a stretched configuration, for the parameters we consider. This is in stark contrast to the situation in the DNA-relay model, where only the initial deflection of the ParA dimer from its equilibrium position when binding to the cargo accounts for the generated force. Another important difference between our model and previously proposed models for the Par system that include the elasticity of the nucleoid [39, 48, 51, 59] is how mobile the ATPase is compared to the cargo. In contrast to the aforementioned Par models, the ATPase (PomZ) diffuses rapidly on the nucleoid and the cargo (Pom cluster) only moves due to its interactions with PomZ dimers, in our model. Fast diffusion of PomZ dimers on the nucleoid and the relatively large spring stiffness of PomZ dimer springs explain the small force exerted on the cluster due to the initial deflection of the spring: this deflection is only small and quickly reduced by diffusion of PomZ. We conclude that force generation based on the elasticity of the nucleoid can be sufficient for cargo translocation even if the mobility of the transporting proteins is higher than the mobility of the cargo.

Our observation of an oscillatory cluster movement when the dynamics of the PomZ dimers is slow compared to the dynamics of the PomXY cluster is in agreement with findings for the Par system [48, 53], despite differences between their models and ours. Similar to our finding that an intermediate ATP hydrolysis rate of PomZ minimizes the time the cluster needs to reach midcell, Hu et al. observed that an intermediate detachment rate of the ATPase from the cargo leads to the most persistent movement of the cargo [59]. However, their model differs from our model as they consider the movement of a fast diffusing cargo on a two-dimensional DNA-carpet to mimic an *in vitro* Par system [47]. In contrast, our model for the *in vivo* Pom system accounts for the nucleoid as an object of finite size. Since the Pom cluster diffuses slowly compared to the PomZ dimers, the diffusive fluxes of PomZ into the cluster need to be accounted for when determining the dependence of the cluster dynamics on the ATP hydrolysis rate (Fig 5).

The model we present here yields a mechanistic understanding of midcell localization of the Pom cluster. So far, not all model parameters are determined experimentally in *M. xanthus* cells. Hence, it would be important to measure the remaining biological rates, such as the nucleoid attachment rate, the diffusion constants and the cluster binding rate *in vivo*. Another limitation of our current model is that it is one-dimensional. How the cluster dynamics changes in a three-dimensional geometry is an interesting question for further research. Furthermore, in the current model we do not account for the PomXY cluster formation, but consider the cluster as a fixed structure. This is motivated by the experimental finding that PomX forms filaments *in vitro* and a high fraction of fluorescently labelled PomX was observed in the cluster *in vivo* [5]. However, it remains unclear how the cluster is formed *in vivo* and how the size of the cluster is maintained from one cell generation to the next.

Our model for the Pom cluster positioning makes three important predictions, which would be interesting to test experimentally: First, the cluster starts to oscillate if PomZ dimers diffuse slowly on the nucleoid. We hypothesize that this might be tested experimentally by increasing the binding affinity of PomZ dimers to the DNA and in this way decreasing the mobility of PomZ on the nucleoid. Second, we predict that there is an optimal ATP hydrolysis rate to minimize the time the cluster takes to reach midnucleoid. Decreasing the rate of ATP hydrolysis by PomZ dimers associated with the PomXY cluster in experiments reduced the velocity of cluster movement towards midcell [5]. It would be interesting to test whether the velocity of the cluster is also reduced for an enhanced ATP hydrolysis rate in *in vivo* experiments. Finally, we predict that the mobility of the PomZ dimers on the Pom cluster can increase the velocity of the cluster movement. To test this model prediction, experiments to uncover the dynamics of PomZ dimers bound to the cluster are needed.

The research presented here gives insights into the dynamics of the Pom cluster in *M. xanthus*, which is determined by its interactions with the nucleoid-bound PomZ dimers. With our semi-analytical approach we gain a better mechanistic understanding of the net force generation in our model. This approach might also prove to be useful for the related ParAB*S* systems or other stochastic, out of equilibrium systems to position intracellular cargoes.

## Materials and methods

The mathematical model is implemented using a Gillespie algorithm [60], a stochastic simulation algorithm. In short, this algorithm works as follows: In each simulation step, all possible actions with their corresponding rates are determined. If the rates are constant in time, the time until any of these actions happens is exponentially distributed with the sum of all rates as rate parameter. To perform one simulation step, a uniformly distributed random number *ξ* ∈ (0, 1] is drawn, which results in a time step

$$\begin{array}{c} \Delta t=-\frac{\ln\xi }{\alpha }, \end{array}$$ (20)

where *α* is the sum over all rates. Then a uniformly distributed random number is drawn to determine which of the possible actions happens. This is done by weighting the different actions according to their rates.

Two different kinds of simulations are performed: In the first, the PomZ dynamics and the PomXY cluster dynamics are simulated (“dynamic cluster simulations”). In the second, the cluster position is kept fixed and only the PomZ dynamics is considered (“stationary cluster simulations”). In the latter case, all rates in the model are constant and the time step for the Gillespie algorithm can be calculated as described above, Eq 20. In contrast, if the PomXY cluster is dynamic, the rates for attachment of a nucleoid-bound PomZ dimer to the PomXY cluster and the hopping rates on the nucleoid, or cluster for cluster-bound PomZ dimers, depend on the cluster position, which is itself time-dependent. The time that elapses before the next action is now given by

$$\begin{array}{c} \int_{t}^{t+\Delta t}\alpha \left(t^{\prime }\right)dt^{\prime }=-\ln\left(\xi \right), \end{array}$$ (21)

which must be solved for Δ*t*. Since an analytical integration of the time-dependent rates is not feasible, the expression needs to be solved numerically, which is computationally costly. However, if the PomXY cluster moves only a small distance between two Gillespie steps, the time-dependent rates also change only slightly. We tested the importance of the time dependence of the rates by approximating the time-dependent rates with their rate at time *t*, and added a rate to the simulation that has no effect, except that the time step preceding the next action is decreased on average. The results obtained when this rate was set to a high value were very similar to those found in its absence. Hence for the parameters we consider in this work, the time dependence of the rates can be ignored.

### Dynamic cluster simulations

In the simulations to determine the cluster dynamics, all PomZ dimers are initially in the cytosol. The PomXY cluster position is kept fixed for *t*_min_ = 10 min with all possible actions of the PomZ dimers allowed. As a result, the initial condition resembles the stationary distribution of PomZ dimers. The initial position of the cluster is such that the left edge of the cluster and the nucleoid coincide.

To derive PomZ flux and density profiles at specific cluster positions, the simulated fluxes and densities are recorded only if the cluster is within a certain distance of a predefined position of interest. For example, to get the PomZ flux / density for clusters at 20% nucleoid length, recording begins when the PomXY cluster is in the region 20 ± 0.2% and stops if it leaves the region 20 ± 1%. Averaging is performed over all times at which the cluster resides within the specific region, weighting each density or flux profile with the corresponding time spent by the cluster in that specific region. To estimate the difference in PomZ flux into the PomXY cluster from either side, the maximal and minimal flux values in the average flux profile of PomZ dimers bound to the nucleoid, but not the PomXY cluster, are determined. These values are typically found a short distance from the edge of the PomXY cluster region, because PomZ dimers can attach to the cluster in a stretched configuration. The two extreme flux values of opposite sign are added together to get the average flux difference of PomZ dimers into the cluster.

#### Analysis of friction coefficient

In the simulations to measure the effective friction coefficient of the PomXY cluster, all PomZ dimers in the system are bound to the PomXY cluster and they cannot detach from it (*k*_*h*_ = 0) such that the number of cluster-bound PomZ dimers is constant. An external force is applied to the cluster and the force-velocity curve is recorded. More specifically, at least three different forces (0.005 pN, 0.01 pN, 0.02 pN) are applied, and the average steady-state cluster velocity is calculated based on 100 trajectories. Plotting the force against the velocity yields a linear dependence, and the friction coefficient can be obtained from the slope. In these simulations an infinitely extended PomXY cluster and nucleoid is considered, i.e. boundaries are neglected. This is done because otherwise the PomZ dimers would accumulate at one of the cluster ends.

#### Analysis of oscillatory properties

In the simulations set up to study the oscillatory behavior of the cluster, the PomXY cluster starts at midnucleoid and its position is recorded over a long time (at least 1000 min). Initially, all PomZ dimers are in the cytosol, but the cluster movement only starts after *t*_min_ = 10 min, such that the PomZ dynamics can approach its stationary distribution. Two observables are of interest: the cluster position distribution and the Fourier spectrum of the cluster trajectories. In the case of the first, the histogram depicting the cluster positions of all runs is smoothed using a Gaussian moving average and peaks are identified in the smoothed profile, which are local maxima or minima. Depending on the parameters chosen, there might be no local minima. In this case, the cluster position distribution has a monomodal shape. If there is a minimum and the difference between the maximal and minimal peak is larger than 2% of the maximal count and the maximum is further away from the midnucleoid position than the minimum, the profile is classified as bimodal.

To determine if the cluster trajectories are oscillatory or not and to estimate the frequencies of cluster oscillations, the procedure used is as follows: For each run, the temporal average of the cluster position is subtracted from the cluster trajectory and a fast Fourier transform of the resulting data is performed. The modulus of the Fourier-transformed cluster position for each run is summed, and the resulting spectrum is smoothed using a moving average with Gaussian weights. Then the largest peak is identified in the smoothed data with a minimal peak height 20% higher than the value corresponding to the smallest frequency, *f*_min_ = 1/*T*_max_, in the smoothed data set (*T*_max_ is the duration of the signal considered in the Fourier transformation). If there is a peak, the cluster trajectory is oscillatory with the frequency determined by the peak in the Fourier spectrum. On the other hand, if no peak is found, the trajectories are classified as “non-oscillatory”.

### Stationary cluster simulations

Simulations with a fixed position of the PomXY cluster are performed to measure the force exerted by a single PomZ dimer on the cluster (“one-particle simulations”) or to measure the PomZ dimer flux into the cluster and the forces exerted on the cluster for an arbitrary number of PomZ dimers in the system. In these simulations, the PomZ dimer(s) are initially in the cytosol. When the adiabatic assumption holds true, the results from the stationary cluster simulations can be used as approximations for the PomZ dynamics in the dynamic cluster simulations.

#### One-particle simulations

To determine the force typically exerted by a single PomZ dimer on the PomXY cluster, simulations with only one PomZ dimer in the system are performed. Here, the PomXY cluster is located far away from the nucleoid boundaries (at midnucleoid) and the PomZ dimer attaches to a lattice site on the right side of the cluster that is so far away from the cluster that no interaction with the cluster is possible. In the simulations, we record the nucleoid and cluster binding site positions when the PomZ dimer attaches to the PomXY cluster, as well as the force exerted on the cluster integrated over time and averaged over time for a number, *N*_runs_, of PomZ dimers interacting with the cluster. To obtain the constant *C* = *f*/*k*_*h*_, the ensemble average of the time-averaged force, *f*, needs to be determined. This quantity is calculated as follows:

$$f=\frac{\sum_{i}f_{i}t_{i}}{\sum_{i}t_{i}}=\frac{\sum_{i}f_{i}^{\text{int}}}{\sum_{i}t_{i}},$$

with *f*_*i*_ and $f_{i}^{\text{int}}$ the time-averaged and time-integrated force exerted by a single PomZ dimer interacting with the PomXY cluster and *t*_*i*_ the corresponding time of interaction. In this definition of *f*, each time-averaged force is weighted by the time the particle remains attached to the PomXY cluster when calculating the mean. Note that the constant *C* can also be expressed in terms of the ensemble average of the time-integrated force, *f*^int^:

$$C=\frac{f}{k_{h}}=\frac{\sum_{i}f_{i}^{\text{int}}}{k_{h}\sum_{i}t_{i}}\approx \frac{\sum_{i}f_{i}^{\text{int}}}{N_{\text{runs}}}=f^{\text{int}},$$

because $\frac{1}{N_{\text{runs}}}\sum_{i}t_{i}=\frac{1}{k_{h}}$ for large *N*_runs_.

## Supporting information

S1 Text Details on the stochastic model for the Pom cluster dynamics. We give details on the stochastic model we introduce in the main text, including a detailed discussion of our choice of parameter values (S1 Table) and our simulation results when detachment of PomZ that is not cluster-bound is incorporated in the model.
(PDF)

S2 Text Details on the RD model. We explain how the parameters in the RD model are defined to match those used in the stochastic simulations. Further, we provide information on the stationary solution of the RD equations.
(PDF)

S3 Text Force a single PomZ dimer exerts on the cluster. We estimate the force a PomZ dimer exerts on the cluster due to binding to it in a stretched configuration in order to estimate which contribution to the force is the most important for the parameters we consider.
(PDF)

S4 Text Derivation of the effective friction coefficient of the PomXY cluster. We derive an analytical expression for the effective friction coefficient of the PomXY cluster, i.e. the friction coefficient when the cluster is tethered to the nucleoid by *N* PomZ dimers.
(PDF)

S1 Table Parameters used in the simulations. If not explicitly stated otherwise the values for the model parameters shown here are those used in the simulations. For a discussion of the parameters see S1 Text.
(PDF)

S1 Fig Midnucleoid localization vs. oscillatory movements. (A-D) The average magnitude of the fast Fourier transform signal (black line) is smoothed using a moving average with Gaussian weights (blue line) to determine whether there is a peak in the Fourier spectrum or not (for details see Materials and methods). The insets show a cluster trajectory for one run. The diffusion constants of PomZ on the nucleoid and PomXY cluster are varied over three orders of magnitudes; the other parameters are chosen as in S1 Table. For the Fourier analysis we performed 100 runs of the simulation for ≥ 1000 min with a cluster starting at midnucleoid.
(PDF)

S2 Fig Additional parameter sweeps. Same as in Fig 2, but here we vary the spring stiffness, *k* (A), and the total number of PomZ dimers, *N*_total_ (B). The spring stiffness can be changed over an order of magnitude without changing the cluster dynamics. However, note that the attachment rate of PomZ dimers to the PomXY cluster is defined in such a way that the total attachment rate to the cluster depends on *k*. The more PomZ dimers are in the system, the faster the clusters move towards midnucleoid.
(PDF)

S3 Fig Cluster dynamics if nucleoid-bound PomZ dimers can also detach from the nucleoid when they are not bound to the cluster. (A) Same as in Fig 2, but here we modified our model described in the main text by allowing PomZ dimers that are bound to the nucleoid to detach (with rate *k*_off_) from the nucleoid into the cytosol also when they do not interact with the PomXY cluster. In black, the simulation results for the model described in the main text (PomZ dimers can only detach when they interact with the cluster) are shown, for comparison reasons. The larger the detachment rate, *k*_off_, the longer it takes until the cluster reaches midnucleoid and for very large detachment rates, the cluster does not reach midnucleoid at all. (B) PomZ flux difference into the cluster as a function of the cluster position for the same detachment rates, *k*_off_, as in A. The black lines indicate the results from the RD model. For the cases with *k*_off_ ≠ 0, we extended the RD equations such that they include detachment of PomZ dimers bound to the nucleoid only. The results from the stochastic simulations (points of different shape and color) nicely agree with the theoretical values. For each parameter set we simulated 100 cluster trajectories.
(PDF)

S4 Fig The net force is proportional to the flux difference for a stationary PomXY cluster. We simulated the PomZ dynamics for a cluster that is kept fixed at different positions on the nucleoid. (A) The PomZ flux difference into the cluster, *j*_diff_, obtained from the simulations (in red) agrees nicely with the predicted flux difference from the RD model (black line). (B) In the simulations, the total force exerted by the PomZ dimers on the PomXY cluster averaged over time, *F*, also decreases towards zero when the cluster is moved from an off-center position towards midnucleoid. (C) The ratio of the total force and the PomZ flux difference (red dots) does not change remarkably with the cluster position, as expected. We discard the value at 50% nucleoid length, because both the flux difference and the total force are supposed to be zero in this case. The black line is a fit of a constant curve to the data with fit parameter *C* = *F*/*j*_diff_ = 0.0059 pN s. The 95% confidence interval of the fit is smaller than the width of the line. The simulated values for the flux difference and the total force are obtained by averaging over 10 realisations of the stochastic simulation per cluster position (the error bars show the 95% confidence interval). The simulation parameters are as in S1 Table.
(PDF)

S5 Fig Force-velocity curve. The average velocity of the PomXY cluster increases linearly with an external force applied to the cluster. For different external force values we simulated 100 trajectories of a PomXY cluster and determined the average steady-state velocity of the cluster (red crosses). A linear fit to the data (black line) matches the simulation results well and yields the effective friction coefficient of the cluster, which is the inverse of the slope. In the simulations an infinitely extended cluster and nucleoid was used (for details see Materials and methods). We simulated *N*_total_ = 20 PomZ dimers, all bound to the PomXY cluster, and the ATP hydrolysis rate *k*_*h*_ was set to zero. The other parameters are as in S1 Table.
(PDF)

S6 Fig Single particle force generation. To determine the constant *C*, simulations with only one PomZ dimer and a fixed PomXY cluster position are performed (parameters as in S1 Table). The PomZ dimer stochastically attaches to the rightmost side of the nucleoid, diffuses on the nucleoid, interacts with the PomXY cluster and then detaches from the PomXY cluster and the nucleoid. We simulated more than 400 000 particle-cluster interactions and recorded the distributions of time-averaged forces (A), time-integrated forces (B) and the distributions of the binding sites of the PomZ dimers on the nucleoid and cluster when attaching to the PomXY cluster (C). The ensemble average of the time-averaged force, weighting each time-averaged force with the corresponding time a PomZ dimer is attached to the cluster, is positive *f* = (5.91 ± 0.02) × 10^−3^ pN (the error is the standard error of the mean). The same holds true for the mean time-integrated force *f*_int_ = (5.92 ± 0.02) × 10^−3^ pN s, which implies that a PomZ dimer arriving at the cluster from the right on average exerts a net force to the right. When attaching to the PomXY cluster, PomZ dimers are typically slightly stretched towards the PomXY cluster, which yields an average distance between the nucleoid and cluster binding site of Δ*x*_0_ ≈ 0.0011 μm.
(PDF)

S7 Fig Friction coefficient of the PomXY cluster. (A-F) We determined the friction coefficient of the PomXY cluster with *N* = 20 PomZ dimers bound to it, when the diffusion constant of PomZ on the nucleoid and the PomXY cluster (A-C), the cytosolic diffusion constant of the PomXY cluster (D), and the spring stiffness of the PomZ dimers (E) is varied. Finally, we varied the PomZ dimer number bound to the PomXY cluster keeping all other parameters fixed (F). In all cases, the friction coefficients obtained from simulations (red dots) agree with the theoretical prediction (black line, Eq 15). The effective friction coefficient of the PomXY cluster increases with an increasing friction of PomZ on the nucleoid and the PomXY cluster, an increasing cytosolic cluster friction and an increasing cluster-bound PomZ dimer number. It does not depend on the spring stiffness of the PomZ dimers for the parameter range considered. For more details see the Materials and methods section. In the simulations performed for this Figure, the nucleoid and PomXY cluster are infinitely extended, all PomZ dimers in the system are bound to the cluster, the ATP hydrolysis rate is set to zero and the other parameters are as in S1 Table if not explicitly given.
(PDF)

S8 Fig Comparison of the average cluster trajectory from simulations and our semi-analytical approximation for additional parameters. Same as in Fig 4, when the spring stiffness *k* (A) and the total PomZ dimer number *N*_total_ (B) is varied. The average cluster trajectories are the same as shown in S2 Fig.
(PDF)

S9 FigForce generation results for additional parameters.Same as in Fig 5 for parameter sweeps varying the attachment rate to the nucleoid *k*_on_, the attachment rate to the PomXY cluster $k_{a}^{0}$ and the spring stiffness *k*. An increase in *k*_on_ and $k_{a}^{0}$ increases the velocity of the cluster towards midnucleoid. An increase in *k* leads to stiffer springs and hence less stretched PomZ dimers, but on the other hand, the force, which is linear in *k*, is increased for the same deflection of the springs. This results in a more or less constant value for *C* and also a constant velocity of the cluster when varying *k* over one order of magnitude. Note that a change in the spring stiffness also changes the total attachment rate of PomZ dimers to the PomXY cluster.(PDF)

S10 Fig Force generation results for additional parameters. Same as in Fig 5 for parameter sweeps varying the diffusion constants of PomZ on the PomXY cluster and the nucleoid (*D*_nuc_ = *D*_clu_), the diffusion constant of PomZ on the nucleoid, *D*_nuc_, and the total PomZ dimer number, *N*_total_. For very small diffusion constants of PomZ on the nucleoid our semi-analytical approach breaks down (see Fig 4). Interestingly, the net velocity of the cluster is maximal for an intermediate diffusion constant of PomZ on the nucleoid and the PomXY cluster, *D*_nuc_ = *D*_clu_ = 0.1 μm^2^/s (see also Fig 4). An increase in the total PomZ dimer number increases the PomZ flux difference into the cluster, but does not change the constant *C*, since *C* is an observable for a single particle. Though the number of PomZ dimers bound to the cluster increases if the total number of PomZ dimers is increased, this does not lead to a significant increase of the friction coefficient of the cluster for the parameters we consider (S1 Table). The velocity of the cluster, which is proportional to the flux difference, then increases with the PomZ dimer number.
(PDF)

S11 Fig Mean time-averaged force for different *k*_*h*_ values. The ensemble average of the time-averaged force a single particle exerts on the PomXY cluster increases with the hydrolysis rate *k*_*h*_. The larger the hydrolysis rate, the shorter the interaction time of the PomZ dimer with the PomXY cluster. Since the PomZ dimers typically attach close to the cluster’s edge and over time diffuse towards the center of the cluster, the average force exerted by the particle decreases over time. Therefore, a shorter interaction time yields a larger time-averaged force. If not explicitly given in the Figure, the parameters are as in S1 Table.
(PDF)

S12 Fig PomZ density and flux for an oscillatory cluster. PomZ density along the nucleoid (A) and PomZ flux difference into the cluster (B) as shown in Fig 3 using the parameters in S1 Table, but a reduced diffusion constant of PomZ on the nucleoid and PomXY cluster (*D*_nuc_ = *D*_clu_ = 0.0001 μm^2^ s^−1^).
(PDF)

S13 Fig Frequency analysis of the cluster dynamics varying *N*_total_ and *L*. The averaged fast Fourier transform of the cluster trajectories and a single trajectory (inset) are shown (see S1 Fig and Materials and methods for details). (A, B) When the total PomZ dimer number is increased from *N*_total_ = 100 to *N*_total_ = 500, the cluster dynamics change from fluctuating around midnucleoid to oscillatory with a frequency of *f* = 0.04 min^−1^ (*D*_nuc_ = *D*_clu_ = 0.01 μm^2^/s, other parameters as in S1 Table). For the Fourier analysis we performed 100 runs of the simulation for 1000 min with a cluster starting at midnucleoid. (C, D) When the nucleoid length, *L*, is increased from *L* = 5 μm to *L* = 15 μm, the peak in the Fourier spectrum, which indicates on average oscillations of the clusters with a frequency *f* = 0.001 min^−1^, disappears (*D*_nuc_, *D*_clu_ = 0.0001 μm^2^/s, other parameters as in S1 Table). We performed 100 runs of the simulation for at least 10 000 min with a cluster starting at midnucleoid.
(PDF)
