# Supplementary material for: Regulation of Pom cluster dynamics in Myxococcus xanthus
Source: PLoS Comput Biol. 2018 Aug 13;14(8):e1006358. doi: 10.1371/journal.pcbi.1006358 (PMC6107250; doi:10.1371/journal.pcbi.1006358)
Supplement: S1 Text — We give details on the stochastic model we introduce in the main text, including a detailed discussion of our choice of parameter values (S1 Table) and our simulation results when detachment of PomZ that is not cluster-bound is incorporated in the model. (PDF) [file pcbi.1006358.s001.pdf]

S1 Text: Details on the stochastic model for the Pom cluster dynamics

1 Is a previously proposed model for a Par positioning system suitable to explain the experimental findings for the Pom system?

Due to the similarities between the Pom system and Par systems for plasmid and chromosome segregation, we thought about whether an existing model for positioning by the Par system might also explain the observed Pom cluster dynamics. However, there is no experimental evidence that PomZ forms filaments [1] and therefore we did not consider filament-based models. Also, several experimental observations in *M. xanthus* cells [2] speak against a diffusion-ratchet mechanism [3–5]: (i) time-lapse experiments show that the Pom cluster is more mobile if PomZ is present than without PomZ, (ii) FRAP experiments indicate that the PomZ dynamics is fast (in the cytosol and on the nucleoid) compared to the cluster dynamics, and (iii) no clear depletion zone in PomZ in the wake of the cluster is visible. The fast PomZ dynamics on the nucleoid is also in contrast to the DNA-relay model [6, 7], where nucleoid-bound ParA dimers are spatially restricted in their movement, similar to a Brownian particle in a trap. In the directed motion model, suggested by Ietswaart et al. [8], ParA dimers can polymerize along the long cell axis and thereby form structures that bias the movement of the plasmids towards equally-spaced positions on the nucleoid. This assumption is in contrast to the Pom system, where no evidence for filament formation is found.

The Pom system also differs from several Par systems by an accumulation of PomZ at the cluster consisting of PomX and PomY proteins and the large cluster size. These experimental observations need to be accounted for in a model for the Pom cluster dynamics. Overall, this necessitates a new model to describe the Pom cluster dynamics.

2 Discussion of the parameters used in the simulations

So far, not all parameters we use in our model are determined experimentally in *M. xanthus*. To nevertheless get an estimate for the physiological values, we approximated the values that are not experimentally determined by the corresponding ones from the related Par system, where possible. Varying the parameters over a broad range, as done in this study, also helps to ensure that the physiological parameter values are included. Importantly, several parameters can be varied without a remarkable change in the cluster dynamics (Fig 2, S2 Fig).

The values for the total number of PomZ dimers, N_{total}, the length of the nucleoid and the cluster, L and L_c, are determined from experiments in *M. xanthus* cells [2]. The rate k_h in our model combines several processes, such as ATP hydrolysis and the conformational change of the PomZ dimer that finally leads to the detachment of PomZ from the nucleoid and the cluster. This rate can be estimated from FRAP experiments in *M. xanthus*. If PomZ is bleached at the position of the PomXY cluster, PomZ dimers recover with a recovery half-time of $(1.2 \pm 0.2)\,\text{s}$ [2]. Based on this time scale for the turnover of PomZ at the cluster, we use $k_h = 1\,\text{s}^{-1}$ in our simulations.

Moreover, the FRAP experiments in [2] indicate that PomZ diffuses quickly on the nucleoid, which can explain the experimentally observed high PomZ density at the cluster although the turnover of cluster-bound PomZ dimers is fast. Based on this observation we chose the diffusion constant of PomZ on the nucleoid to $D_{\text{nuc}} = 0.1\,\mu\text{m}^2/\text{s}$, which is in the upper range of

values reported in the literature for ParA dimer diffusion on the nucleoid (from $0.001\,\mu m^2/s$ [5] to $1\,\mu m^2/s$ [8,9]). Since the dynamics of PomZ dimers bound to the cluster is not measured yet, we set the diffusion constant of PomZ dimers on the PomXY cluster, D_{clu}, to the same value as D_{nuc}.

The attachment rate of PomZ to the nucleoid, k_{on}, combines several biochemical processes: Before PomZ that was just released at the cluster can rebind to the nucleoid, it needs to exchange ADP for ATP, dimerize and regain the ability to bind non-specific DNA [2,10]. *In vitro* measurements of the DNA binding rate of ParA that can bind non-specifically to DNA [10] suggest a binding rate of about $50\,s^{-1}$. Since it takes long for ParA to regain the DNA binding ability compared to the binding itself [10] and they have to reach the nucleoid before they can bind to it, this value can be regarded as an upper bound. In previous models for the ParA positioning system, the rate for ParA to rebind the nucleoid has been chosen between $0.01\,s^{-1}$–$50\,s^{-1}$ [5–8]. We used a value in this range, $k_{\mathrm{on}} = 0.1\,s^{-1}$, and varied the rate two orders of magnitude in our simulations. We find that above a certain value of k_{on} the trajectories do not change remarkably if the rate is increased even more (Fig 2). The same holds true for the attachment rate of PomZ dimers to the PomXY cluster, k_a^0. We chose the attachment rate of PomZ to the cluster such that the cluster dynamics does not change remarkably if k_a^0 is increased.

The friction coefficient of the cluster, $\gamma_c = k_B T / D_{\mathrm{cluster}}$, we used as a fit parameter to obtain the experimentally observed time scale for the clusters to reach midcell of about 80 min, in our simulations. The fit result, $D_{\mathrm{cluster}} \approx 0.0004\,\mu m^2/s$, is comparable to literature values for plasmids ($0.001\,\mu m^2/s$ [7,8]), though a bit smaller. A smaller diffusion constant of the Pom cluster compared to plasmids is expected because of the large size of the cluster. The effective spring stiffness, k, which accounts for the elasticity of the nucleoid and the PomZ dimers, is approximated by the value for the stiffness of a bond between the plasmid and the nucleoid via ParA dimers used in [5]. To test our choice of the lattice spacing, we also performed simulations using the parameters as in S1 Table, but with $a = 0.005\,\mu m$. We did not observe remarkable changes in the cluster dynamics compared to our results with $a = 0.01\,\mu m$.

3 Simulations with detachment of nucleoid-, but not cluster-bound PomZ

In the stochastic model discussed in the main text we assume that PomZ dimers only detach from the nucleoid into the cytosol, when they are also bound to the cluster. This assumption is motivated by the following experimental observations: A mutant in PomZ that does not hydrolyze ATP (PomZ-D90A) colocalizes with the nucleoid if the cluster is absent and with the cluster if it is present [2], which shows that detachment of PomZ dimers from the nucleoid is ATP-dependent. The ATP turnover rate for PomZ in contact with DNA alone is small compared to that of PomZ in contact with PomX, PomY and DNA. Together, these observations suggest that detachment of PomZ from the nucleoid away from the cluster is a minor contribution compared to detachment of cluster-bound PomZ [2].

Although detachment of PomZ away from the cluster might occur only rarely, it could have an effect on the cluster dynamics. To investigate this effect, we simulated cluster trajectories in a model identical to the one discussed in the main text, but which also includes detachment of PomZ dimers that are bound to the nucleoid only. The simulation results (S3 Fig) show that the larger the detachment rate, k_{off}, the longer the clusters need to reach midcell and for very large rates the cluster does not reach midcell at all. We expect that if the typical

length a PomZ dimer diffuses on the nucleoid before it detaches, $L_{\text{diff}} = \sqrt{2D_{\text{nuc}}/k_{\text{off}}}$, is larger than $(L - L_c)/2$, the cluster is positioned at midcell. This condition implies that the detachment rate, k_{off}, needs to be smaller than $8D_{\text{nuc}}/(L - L_c)^2 \approx 0.04\,\text{s}^{-1}$ for midnucleoid positioning. Indeed, our simulation results show midcell localization for detachment rates below this threshold. Even for detachment rates above the threshold, $k_{\text{off}} = 0.1\,\text{s}^{-1}$, midcell localization of the cluster is obtained (S3 Fig). How the detachment rate influences the cluster dynamics can be understood in more detail by considering the flux difference of PomZ dimers into the cluster, which decreases with an increasing detachment rate (S3 Fig). For very large detachment rates the flux difference of PomZ dimers into the cluster vanishes already before the cluster is at midcell, leading to the stalling of the cluster at an off-center position observed in the simulations (S3 Fig).

Supplementary references

1. Treuner-Lange A, Aguiluz K, van der Does C, Gómez-Santos N, Harms A, Schumacher D, et al. PomZ, a ParA-like protein, regulates Z-ring formation and cell division in *Myxococcus xanthus*. Mol Microbiol. 2013;87(2):235–253. doi:10.1111/mmi.12094.

2. Schumacher D, Bergeler S, Harms A, Vonck J, Huneke-Vogt S, Frey E, et al. The PomXYZ proteins self-organize on the bacterial nucleoid to stimulate cell division. Dev Cell. 2017;41(3):299–314. doi:10.1016/j.devcel.2017.04.011.

3. Vecchiarelli AG, Neuman KC, Mizuuchi K. A propagating ATPase gradient drives transport of surface-confined cellular cargo. Proc Natl Acad Sci U S A. 2014;111(13):4880–4885. doi:10.1073/pnas.1401025111.

4. Hu L, Vecchiarelli AG, Mizuuchi K, Neuman KC, Liu J. Directed and persistent movement arises from mechanochemistry of the ParA/ParB system. Proc Natl Acad Sci U S A. 2015;112(51):E7055–E7064. doi:10.1073/pnas.1505147112.

5. Hu L, Vecchiarelli AG, Mizuuchi K, Neuman KC, Liu J. Brownian ratchet mechanism for faithful segregation of low-copy-number plasmids. Biophys J. 2017;112(7):1489–1502. doi:10.1016/j.bpj.2017.02.039.

6. Lim HC, Surovtsev IV, Beltran BG, Huang F, Bewersdorf J, Jacobs-Wagner C. Evidence for a DNA-relay mechanism in ParABS-mediated chromosome segregation. Elife. 2014;3:e02758. doi:10.7554/eLife.02758.

7. Surovtsev IV, Campos M, Jacobs-Wagner C. DNA-relay mechanism is sufficient to explain ParA-dependent intracellular transport and patterning of single and multiple cargos. Proc Natl Acad Sci U S A. 2016;113(46):E7268–E7276. doi:10.1073/pnas.1616118113.

8. Ietswaart R, Szardenings F, Gerdes K, Howard M. Competing ParA structures space bacterial plasmids equally over the nucleoid. PLoS Comput Biol. 2014;10(12). doi:10.1371/journal.pcbi.1004009.

9. Vecchiarelli AG, Hwang LC, Mizuuchi K. Cell-free study of F plasmid partition provides evidence for cargo transport by a diffusion-ratchet mechanism. Proc Natl Acad Sci U S A. 2013;110(15). doi:10.1073/pnas.1302745110.

10. Vecchiarelli AG, Han YW, Tan X, Mizuuchi M, Ghirlando R, Biertümpfel C, et al. ATP control of dynamic P1 ParA-DNA interactions: a key role for the nucleoid in plasmid partition. Mol Microbiol. 2010;78(1):78–91. doi:10.1111/j.1365-2958.2010.07314.x.
