# Supplementary material for: Regulation of Pom cluster dynamics in Myxococcus xanthus
Source: PLoS Comput Biol. 2018 Aug 13;14(8):e1006358. doi: 10.1371/journal.pcbi.1006358 (PMC6107250; doi:10.1371/journal.pcbi.1006358)
Supplement: S2 Text — We explain how the parameters in the RD model are defined to match those used in the stochastic simulations. Further, we provide information on the stationary solution of the RD equations. (PDF) [file pcbi.1006358.s002.pdf]

S2 Text: Details on the RD Model

1 Parameters in the RD model

In the following we explain how we chose the parameters in the RD model to make it comparable to our stochastic model.

Attachment rate of PomZ to the cluster. To obtain an expression for k_a^{total} that resembles attachment in the stochastic model, we need to integrate the rate of attachment of PomZ dimers positioned at x on the nucleoid to position y on the PomXY cluster over all possible cluster binding site positions

$$k_a^{\text{total}}(x) = \int_{x_c - L_c/2}^{x_c + L_c/2} k_a^0 e^{-\frac{1}{2}\frac{k}{k_B T}(y-x)^2} dy,$$

with L_c the length of the cluster and x_c the cluster position. The spring stiffness is denoted by k and the rate k_a^0 is the attachment rate of nucleoid-bound PomZ dimers to the PomXY cluster (in an unstretched configuration) per unit of time and length. Since we expect the physiological value of the spring stiffness k to be large (S1 Table, [1]), the Boltzmann factor decays quickly, and hence we can neglect boundary effects of the PomXY cluster ($L_c \to \infty$). With this we obtain the following approximation for k_a^{total} used in the RD model:

$$k_a^{\text{total}} = \begin{cases} k_a^0 \sqrt{\frac{2\pi k_B T}{k}}, & x \in I_c, \\ 0, & \text{otherwise.} \end{cases}$$

Diffusion constant of PomZ bound to the nucleoid and cluster. In the RD model we assume that PomZ dimers bound to the PomXY cluster and the nucleoid diffuse with a diffusion constant D_b. How does this value depend on the diffusion constants of PomZ on the nucleoid and on the PomXY cluster, D_{nuc} and D_{clu}, and on the spring stiffness, k, in the stochastic model? If the diffusion constants on the cluster and the nucleoid are the same, $D_{\text{clu}} = D_{\text{nuc}} \equiv D$, the center of mass of the PomZ dimer diffuses with $D_b = 0.5D$, independently of the spring stiffness. This result is known from the Rouse model, which models polymers as n beads coupled by springs. Here, the diffusion constant of the center of mass of the polymer decreases with the number of beads as $1/n$, which results in the factor of 0.5 in the case of two beads [2]. For a stiff spring, coupling the nucleoid and cluster binding sites, the diffusion constant of the two binding sites can be considered as equal and equivalent to that for the center of mass. Based on these considerations we approximate the diffusion constant D_b in the RD model by $D_b = 0.5\, D_{\text{nuc}}$.

Note that a large spring stiffness is necessary for the good agreement between the RD and the stochastic model (see Fig 3), because in the RD model we do not model the PomZ dimers as springs (as in the stochastic model), which is best reflected by a large spring stiffness.

2 Stationary solution of the RD model

We solved the reaction-diffusion equations (Eq 9-14 in the main text) in the steady-state to get analytical expressions for the flux difference of PomZ dimers into the cluster and the number of PomZ dimers bound to the PomXY cluster. Eqs 9 and 10 are solved by imposing

no flux boundary conditions at $x = 0$ and $x = L$ and setting the values for the concentration, $c(x)$, and its derivative, $\partial_x c(x)$, inside and outside of the PomXY cluster region equal at the cluster boundaries, $x = x_c \pm L_c/2$. The second condition, an equivalence of the first derivative of the concentration, is due to balance of diffusive fluxes at $x = x_c \pm L_c/2$. Since the diffusion constants of PomZ dimers bound to the nucleoid, but not bound to the PomXY cluster, are the same in the cluster region and outside of it, the first derivative has to be set equal at $x = x_c \pm L_c/2$. The resulting expression for $c(x)$ can be used to solve Eq 11 in the steady state. For the cluster-bound PomZ dimers, $c_b(x)$, no-flux boundary conditions hold at the PomXY cluster's edges. The last unknown is N_{cyto}, which is determined by mass conservation,

$$\int_0^L c(x)\,dx + \int_{x_c-L_c/2}^{x_c+L_c/2} c_b(x)\,dx + N_{\text{cyto}} = N_{\text{total}},$$

or equivalently by solving Eq 12 in the steady state. The solutions for $c(x), c_b(x)$ and N_{cyto} are obtained with *Mathematica* [3]. They are quite lengthy and hence not written out here explicitly. The flux difference of PomZ dimers into the cluster can then be calculated as follows:

$$j_{\text{diff}}(x_c) = D_{\text{nuc}} \partial_x c(x)|_{x_c+L_c/2} + D_{\text{nuc}} \partial_x c(x)|_{x_c-L_c/2}.$$

We chose the sign of the flux difference to be positive if more PomZ dimers arrive from the right than from the left side. The number of PomZ dimers bound to the PomXY cluster is given by:

$$N(x_c) = \int_{x_c-L_c/2}^{x_c+L_c/2} c_b(x) dx.$$

Supplementary references

1. Hu L, Vecchiarelli AG, Mizuuchi K, Neuman KC, Liu J. Brownian ratchet mechanism for faithful segregation of low-copy-number plasmids. Biophys J. 2017;112(7):1489–1502. doi:10.1016/j.bpj.2017.02.039.

2. De Gennes PG. Dynamics of entangled polymer solutions. I. The Rouse model. Macromolecules. 1976;9(4):587–593. doi:10.1021/ma60052a011.

3. Wolfram Research, Inc., Mathematica, Version 11.1, Champaign, IL. 2017.
