# Supplementary material for: Regulation of Pom cluster dynamics in Myxococcus xanthus
Source: PLoS Comput Biol. 2018 Aug 13;14(8):e1006358. doi: 10.1371/journal.pcbi.1006358 (PMC6107250; doi:10.1371/journal.pcbi.1006358)
Supplement: S3 Text — We estimate the force a PomZ dimer exerts on the cluster due to binding to it in a stretched configuration in order to estimate which contribution to the force is the most important for the parameters we consider. (PDF) [file pcbi.1006358.s003.pdf]

S3 Text: Force a single PomZ dimer exerts on the cluster

A PomZ dimer can exert a net force on the PomXY cluster when it attaches in a stretched configuration and when it encounters the cluster's edge. In principle, PomZ dimers can also generate a net force due to the reflecting boundary conditions at the nucleoid ends, which leads to a movement of the cluster towards midnucleoid. However, the latter can only represent a minor contribution, since PomZ dimers rarely encounter the nucleoid ends.

To investigate which contribution to the force is most important for the parameters we consider, we estimate the force that is exerted on the cluster due to the initial deflection of the PomZ dimers when binding to the cluster. We do so by assuming that the cluster binding site is fixed. In this case, the movement of the nucleoid binding site can be considered as an Ornstein-Uhlenbeck process, i.e. a particle diffusing in a potential given by the energy of the spring. The time-integrated force then reads

$$f_{\text{int}}^{\text{OU}} = \int_0^{1/k_h} k \Delta x_0 e^{-\beta k D_{\text{nuc}} t} dt \approx \frac{\Delta x_0}{\beta D_{\text{nuc}}} \approx 4.4 \times 10^{-5}\,\text{pN s}.$$

Here we used that the time scale for relaxation in the Ornstein-Uhlenbeck process, $\tau_{\text{OU}} = 1/(\beta k D_{\text{nuc}}) = 0.001\,\text{s}$, is much less than the time for which a PomZ dimer is typically attached to the PomXY cluster, $\tau = 1/k_h = 1\,\text{s}$, for the parameter set in S1 Table. The average distance between the nucleoid and cluster binding site when PomZ attaches to the cluster is obtained from one-particle simulations, $\Delta x_0 \approx 0.0011\,\mu\text{m}$. We find that f_{int}^{OU} is two orders of magnitudes smaller than the ensemble average of the time-integrated forces obtained from the simulations, $f_{\text{int}} = (5.92 \pm 0.02) \times 10^{-3}\,\text{pN s}$ (the error denotes the standard error of the mean). Hence, the force produced due to the initial deflection of the PomZ dimer is not the main contribution to the time-integrated force for the parameters considered here. Instead, the force exerted by a PomZ dimer when it encounters the cluster's edge is an important contribution to the net force generated.
