# Supplementary material for: Regulation of Pom cluster dynamics in Myxococcus xanthus
Source: PLoS Comput Biol. 2018 Aug 13;14(8):e1006358. doi: 10.1371/journal.pcbi.1006358 (PMC6107250; doi:10.1371/journal.pcbi.1006358)
Supplement: S4 Text — We derive an analytical expression for the effective friction coefficient of the PomXY cluster, i.e. the friction coefficient when the cluster is tethered to the nucleoid by N PomZ dimers. (PDF) [file pcbi.1006358.s004.pdf]

# S4 Text: Derivation of the effective friction coefficient of the PomXY cluster

Our aim is to get an analytical expression for the effective friction coefficient of the PomXY cluster, i.e. the friction coefficient when the cluster is tethered to the nucleoid by $N$ PomZ dimers. We consider an infinitely extended PomXY cluster and nucleoid to exclude boundary effects. Note that the absolute positions of the PomZ dimers do not matter, only the difference between the positions of the nucleoid and cluster binding sites matters. Hence, all PomZ dimers can be moved to the same nucleoid position. The position of the cluster binding site then has a distribution that is peaked at the position of the nucleoid binding site if no force is exerted to the PomXY cluster and the peak of the distribution is shifted to the right if the PomXY cluster is pulled to the right by an external force. Let us denote the position of the cluster by $x(t)$. The position of the nucleoid binding site of all PomZ dimers is denoted $x^{\text{nuc}}(t)$, and the average position of the cluster binding sites $x(t) + \Delta x^{\text{clu}}(t)$. Then the following equations hold

$$\begin{aligned}
\partial_t x(t) &= \frac{F}{\gamma_c} - N\frac{k}{\gamma_c}(x(t) + \Delta x^{\text{clu}}(t) - x^{\text{nuc}}(t)),\\
\partial_t \Delta x^{\text{clu}}(t) &= -\frac{k}{\gamma_{\text{clu}}}(x(t) + \Delta x^{\text{clu}}(t) - x^{\text{nuc}}(t)),\\
\partial_t x^{\text{nuc}}(t) &= -\frac{k}{\gamma_{\text{nuc}}}(-x(t) - \Delta x^{\text{clu}}(t) + x^{\text{nuc}}(t)),
\end{aligned}$$

with $\gamma_{\text{clu}}$, $\gamma_{\text{nuc}}$ the friction coefficient of PomZ dimers on the PomXY cluster and the nucleoid, respectively. They are related to the diffusion constants of a PomZ dimer on the nucleoid and PomXY cluster via Stokes-Einstein, $D_{\text{clu/nuc}} = k_B T/\gamma_{\text{clu/nuc}}$. We solved the coupled ODE system above using *Mathematica* [1]. Taking the time derivative of the position of the PomXY cluster, $x(t)$, and dividing $F$ by this expression for the velocity of the cluster yields the effective friction coefficient of the PomXY cluster in dependence of $N$ and the other model parameters:

$$\gamma(t, N) = \frac{e^{k(1/\gamma_{\text{clu}}+1/\gamma_{\text{nuc}}+N/\gamma_c)t}\gamma_c(\gamma_c(\gamma_{\text{clu}} + \gamma_{\text{nuc}}) + \gamma_{\text{clu}}\gamma_{\text{nuc}}N)}{e^{k(1/\gamma_{\text{clu}}+1/\gamma_{\text{nuc}}+N/\gamma_c)t}\gamma_c(\gamma_{\text{clu}} + \gamma_{\text{nuc}}) + \gamma_{\text{clu}}\gamma_{\text{nuc}}N}.$$

For large times ($t \to \infty$), this simplifies to

$$\gamma(N) = \gamma_c + \frac{\gamma_{\text{clu}}\gamma_{\text{nuc}}N}{\gamma_{\text{clu}} + \gamma_{\text{nuc}}}. \tag{S1}$$

We find that the effective friction coefficient of the cluster is given by the cytosolic friction plus an additional term that increases linearly with the number of PomZ dimers bound to the cluster, $N$. This expression is a generalization of the term derived by Lansky et al., [2] using a force-balance argument. The simulations fit well with this theoretical curve if we use an infinitely extended nucleoid and PomXY cluster, i.e. we neglect the boundary conditions (S7 Fig). If the nucleoid and PomXY cluster size is finite in the simulations, the measured effective friction coefficient is higher than the analytical result derived here, because the PomZ dimers move to the rear cluster's edge when the cluster is pulled forward and the

reflecting boundary conditions of the cluster for the movement of PomZ's cluster binding site increase the friction. However, in the dynamic cluster simulations, i.e. simulations with a finite nucleoid and PomXY cluster, which is moved on the nucleoid by PomZ dimer interactions, cluster-bound PomZ dimers can detach into the cytosol with the ATP hydrolysis rate $k_h$. Hence, the effect of accumulation of PomZ dimers at the rear cluster's edge is diminished. In these simulations, the number of cluster-bound PomZ dimers depends on the position of the cluster on the nucleoid. In this case, we replace Eq S1 with

$$\gamma(x_c) = \gamma_c + \frac{\gamma_{\text{clu}}\gamma_{\text{nuc}}N(x_c)}{\gamma_{\text{clu}} + \gamma_{\text{nuc}}}.$$

## Supplementary references


1. Wolfram Research, Inc., Mathematica, Version 11.1, Champaign, IL. 2017.

2. Lansky Z, Braun M, Lüdecke A, Schlierf M, ten Wolde PR, Janson ME, et al. Diffusible crosslinkers generate directed forces in microtubule networks. Cell. 2015;160:1159–1168. doi:10.1016/j.cell.2015.01.051.
