# Supplementary material for: Regulation of Pom cluster dynamics in Myxococcus xanthus
Source: PLoS Comput Biol. 2018 Aug 13;14(8):e1006358. doi: 10.1371/journal.pcbi.1006358 (PMC6107250; doi:10.1371/journal.pcbi.1006358)
Supplement: S1 Table — If not explicitly stated otherwise the values for the model parameters shown here are those used in the simulations. For a discussion of the parameters see S1 Text. (PDF) [file pcbi.1006358.s005.pdf]

## S1 Table: Parameters used in the simulations

| Parameter | Symbol | Value |
|---|---|---|
| Number of PomZ dimers | $N_{\text{total}}$ | $100^*$ |
| Length of nucleoid | $L$ | $5\,\mu\text{m}^*$ |
| Length of cluster | $L_c$ | $0.7\,\mu\text{m}$ |
| Attachment rate PomZ to nucleoid | $k_{\text{on}}$ | $0.1\,\text{s}^{-1}$ |
| Attachment rate PomZ to PomXY cluster (unstretched) | $k_a^0$ | $500\,\text{s}^{-1}\,\mu\text{m}^{-1}$ |
| Diffusion constant PomZ on nucleoid | $D_{\text{nuc}}$ | $0.1\,\mu\text{m}^2/\text{s}$ |
| Diffusion constant PomZ on PomXY cluster | $D_{\text{clu}}$ | $0.1\,\mu\text{m}^2/\text{s}$ |
| ATP hydrolysis rate PomZ bound to PomXY cluster | $k_h$ | $1\,\text{s}^{-1}$ |
| Diffusion constant PomXY cluster in cytosol | $D_{\text{cluster}} = k_B T/\gamma_c$ | $0.0004\,\mu\text{m}^2/\text{s}$ |
| Spring constant PomZ dimers | $k$ | $10^4\,\text{k}_\text{B}\text{T}/\mu\text{m}^2$ |
| Lattice spacing | $a$ | $0.01\,\mu\text{m}$ |

* In the one-particle simulations, the total number of PomZ dimers, $N_{\text{total}}$, is one and the nucleoid length is chosen to be $L = 2.1\,\mu\text{m}$ to decrease computation time.
