# Supplementary material for: Regulation of Pom cluster dynamics in Myxococcus xanthus
Source: PLoS Comput Biol. 2018 Aug 13;14(8):e1006358. doi: 10.1371/journal.pcbi.1006358 (PMC6107250; doi:10.1371/journal.pcbi.1006358)
Supplement: S1 Fig — (A-D) The average magnitude of the fast Fourier transform signal (black line) is smoothed using a moving average with Gaussian weights (blue line) to determine whether there is a peak in the Fourier spectrum or not (for details see Materials and methods). The insets show a cluster trajectory for one run. The diffusion constants of PomZ on the nucleoid and PomXY cluster are varied over three orders of magnitudes; the other parameters are chosen as in S1 Table. For the Fourier analysis we performed 100 runs of the simulation for ≥ 1000 min with a cluster starting at midnucleoid. (PDF) [file pcbi.1006358.s006.pdf]

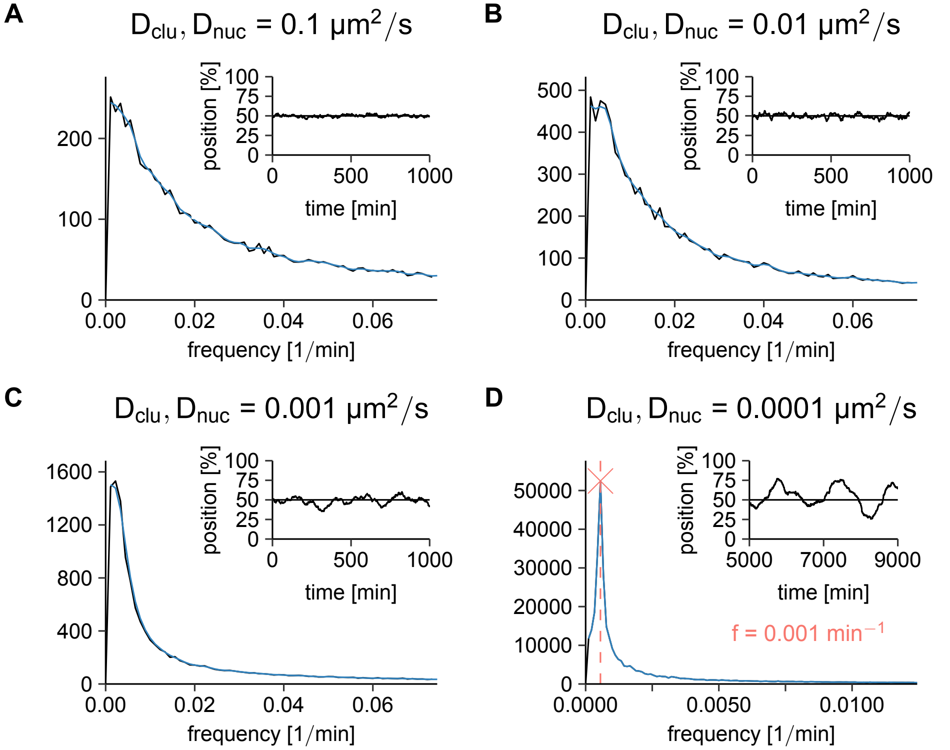
A
D_{clu}, D_{nuc} = 0.1 μm²/s
B
D_{clu}, D_{nuc} = 0.01 μm²/s
C
D_{clu}, D_{nuc} = 0.001 μm²/s
D
D_{clu}, D_{nuc} = 0.0001 μm²/s
position [%]
time [min]
frequency [1/min]
f = 0.001 min⁻¹
