# Supplementary figures and images for: Regulation of Pom cluster dynamics in Myxococcus xanthus
Source: PLoS Comput Biol. 2018 Aug 13;14(8):e1006358. doi: 10.1371/journal.pcbi.1006358 (PMC6107250; doi:10.1371/journal.pcbi.1006358)

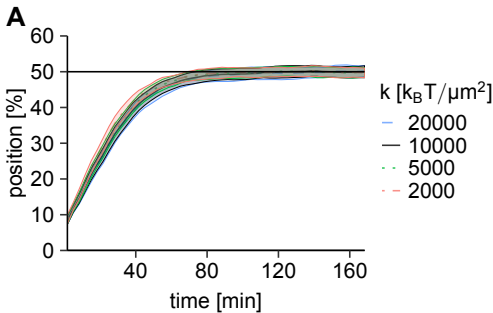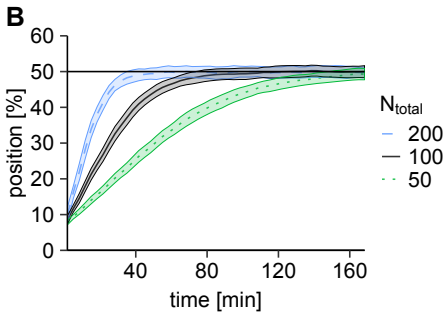

Supplement: S2 Fig — Same as in Fig 2, but here we vary the spring stiffness, k (A), and the total number of PomZ dimers, Ntotal (B). The spring stiffness can be changed over an order of magnitude without changing the cluster dynamics. However, note that the attachment rate of PomZ dimers to the PomXY cluster is defined in such a way that the total attachment rate to the cluster depends on k. The more PomZ dimers are in the system, the faster the clusters move towards midnucleoid. (PDF) [file pcbi.1006358.s007.pdf]

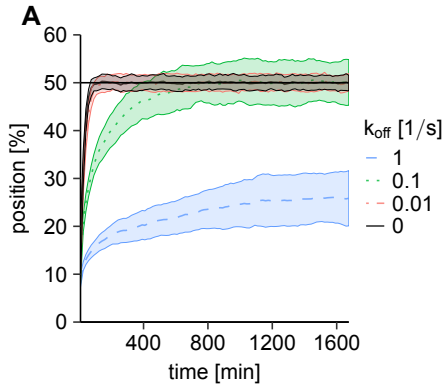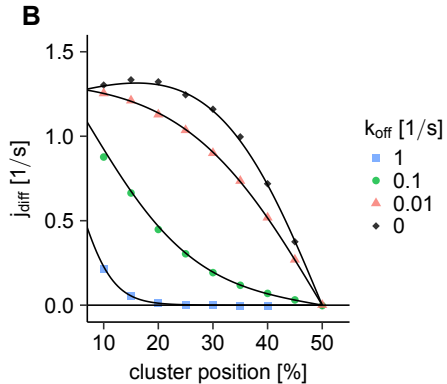

Supplement: S3 Fig — (A) Same as in Fig 2, but here we modified our model described in the main text by allowing PomZ dimers that are bound to the nucleoid to detach (with rate koff) from the nucleoid into the cytosol also when they do not interact with the PomXY cluster. In black, the simulation results for the model described in the main text (PomZ dimers can only detach when they interact with the cluster) are shown, for comparison reasons. The larger the detachment rate, koff, the longer it takes until the cluster reaches midnucleoid and for very large detachment rates, the cluster does not reach midnucleoid at all. (B) PomZ flux difference into the cluster as a function of the cluster position for the same detachment rates, koff, as in A. The black lines indicate the results from the RD model. For the cases with koff ≠ 0, we extended the RD equations such that they include detachment of PomZ dimers bound to the nucleoid only. The results from the stochastic simulations (points of different shape and color) nicely agree with the theoretical values. For each parameter set we simulated 100 cluster trajectories. (PDF) [file pcbi.1006358.s008.pdf]

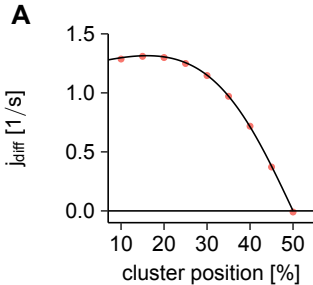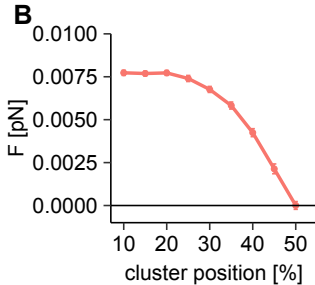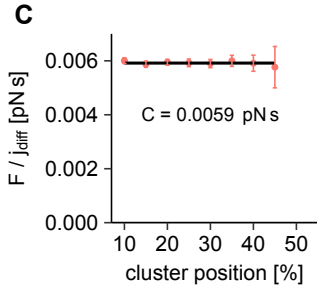

Supplement: S4 Fig — We simulated the PomZ dynamics for a cluster that is kept fixed at different positions on the nucleoid. (A) The PomZ flux difference into the cluster, jdiff, obtained from the simulations (in red) agrees nicely with the predicted flux difference from the RD model (black line). (B) In the simulations, the total force exerted by the PomZ dimers on the PomXY cluster averaged over time, F, also decreases towards zero when the cluster is moved from an off-center position towards midnucleoid. (C) The ratio of the total force and the PomZ flux difference (red dots) does not change remarkably with the cluster position, as expected. We discard the value at 50% nucleoid length, because both the flux difference and the total force are supposed to be zero in this case. The black line is a fit of a constant curve to the data with fit parameter C = F/jdiff = 0.0059 pN s. The 95% confidence interval of the fit is smaller than the width of the line. The simulated values for the flux difference and the total force are obtained by averaging over 10 realisations of the stochastic simulation per cluster position (the error bars show the 95% confidence interval). The simulation parameters are as in S1 Table. (PDF) [file pcbi.1006358.s009.pdf]

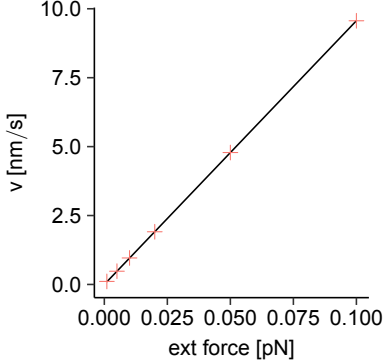

Supplement: S5 Fig — The average velocity of the PomXY cluster increases linearly with an external force applied to the cluster. For different external force values we simulated 100 trajectories of a PomXY cluster and determined the average steady-state velocity of the cluster (red crosses). A linear fit to the data (black line) matches the simulation results well and yields the effective friction coefficient of the cluster, which is the inverse of the slope. In the simulations an infinitely extended cluster and nucleoid was used (for details see Materials and methods). We simulated Ntotal = 20 PomZ dimers, all bound to the PomXY cluster, and the ATP hydrolysis rate kh was set to zero. The other parameters are as in S1 Table. (PDF) [file pcbi.1006358.s010.pdf]

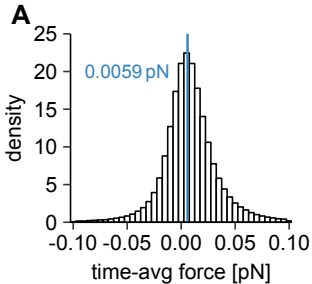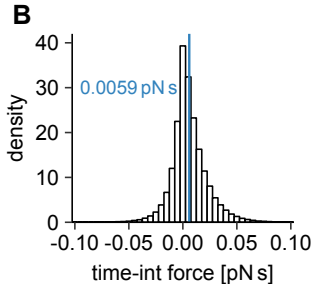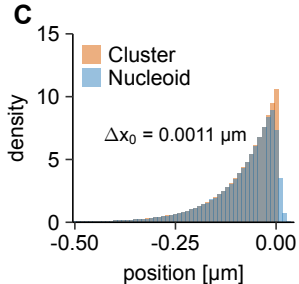

Supplement: S6 Fig — To determine the constant C, simulations with only one PomZ dimer and a fixed PomXY cluster position are performed (parameters as in S1 Table). The PomZ dimer stochastically attaches to the rightmost side of the nucleoid, diffuses on the nucleoid, interacts with the PomXY cluster and then detaches from the PomXY cluster and the nucleoid. We simulated more than 400 000 particle-cluster interactions and recorded the distributions of time-averaged forces (A), time-integrated forces (B) and the distributions of the binding sites of the PomZ dimers on the nucleoid and cluster when attaching to the PomXY cluster (C). The ensemble average of the time-averaged force, weighting each time-averaged force with the corresponding time a PomZ dimer is attached to the cluster, is positive f = (5.91 ± 0.02) × 10−3 pN (the error is the standard error of the mean). The same holds true for the mean time-integrated force fint = (5.92 ± 0.02) × 10−3 pN s, which implies that a PomZ dimer arriving at the cluster from the right on average exerts a net force to the right. When attaching to the PomXY cluster, PomZ dimers are typically slightly stretched towards the PomXY cluster, which yields an average distance between the nucleoid and cluster binding site of Δx0 ≈ 0.0011 μm. (PDF) [file pcbi.1006358.s011.pdf]

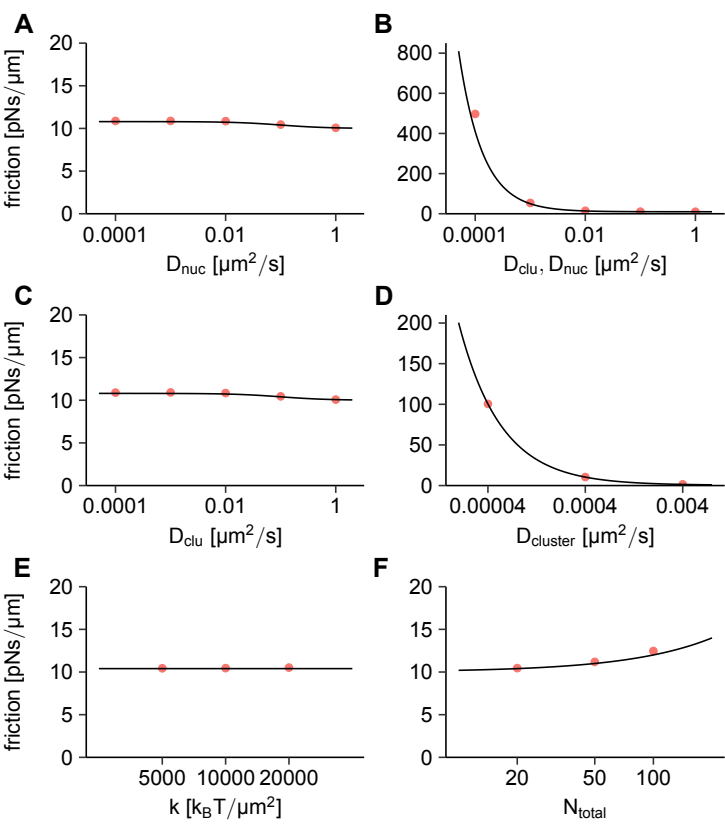

Supplement: S7 Fig — (A-F) We determined the friction coefficient of the PomXY cluster with N = 20 PomZ dimers bound to it, when the diffusion constant of PomZ on the nucleoid and the PomXY cluster (A-C), the cytosolic diffusion constant of the PomXY cluster (D), and the spring stiffness of the PomZ dimers (E) is varied. Finally, we varied the PomZ dimer number bound to the PomXY cluster keeping all other parameters fixed (F). In all cases, the friction coefficients obtained from simulations (red dots) agree with the theoretical prediction (black line, Eq 15). The effective friction coefficient of the PomXY cluster increases with an increasing friction of PomZ on the nucleoid and the PomXY cluster, an increasing cytosolic cluster friction and an increasing cluster-bound PomZ dimer number. It does not depend on the spring stiffness of the PomZ dimers for the parameter range considered. For more details see the Materials and methods section. In the simulations performed for this Figure, the nucleoid and PomXY cluster are infinitely extended, all PomZ dimers in the system are bound to the cluster, the ATP hydrolysis rate is set to zero and the other parameters are as in S1 Table if not explicitly given. (PDF) [file pcbi.1006358.s012.pdf]

**A**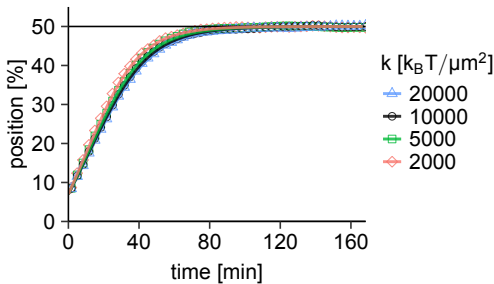**B**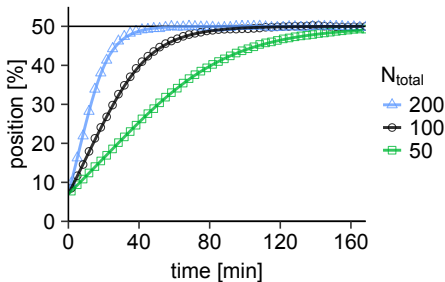

Supplement: S8 Fig — Same as in Fig 4, when the spring stiffness k (A) and the total PomZ dimer number Ntotal (B) is varied. The average cluster trajectories are the same as shown in S2 Fig. (PDF) [file pcbi.1006358.s013.pdf]

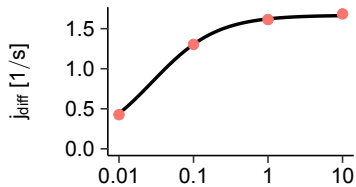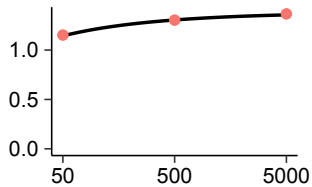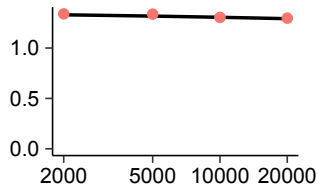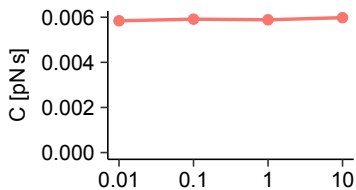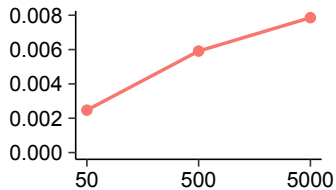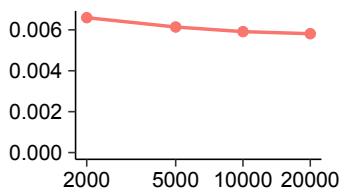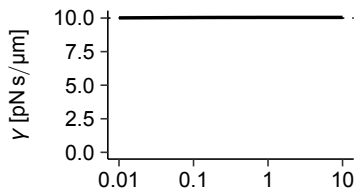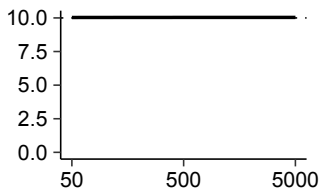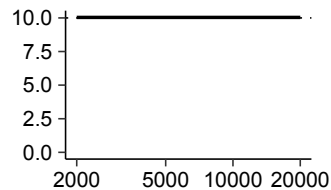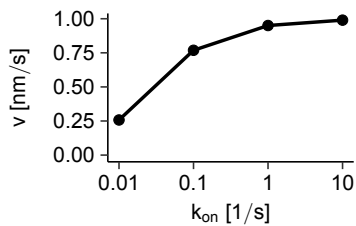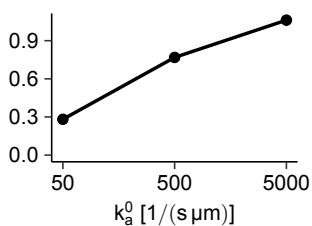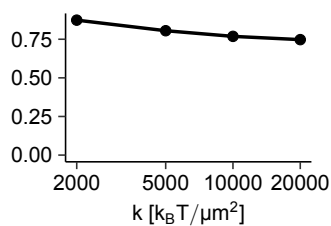

Supplement: S9 Fig — Same as in Fig 5 for parameter sweeps varying the attachment rate to the nucleoid kon, the attachment rate to the PomXY cluster ka0 and the spring stiffness k. An increase in kon and ka0 increases the velocity of the cluster towards midnucleoid. An increase in k leads to stiffer springs and hence less stretched PomZ dimers, but on the other hand, the force, which is linear in k, is increased for the same deflection of the springs. This results in a more or less constant value for C and also a constant velocity of the cluster when varying k over one order of magnitude. Note that a change in the spring stiffness also changes the total attachment rate of PomZ dimers to the PomXY cluster. (PDF) [file pcbi.1006358.s014.pdf]

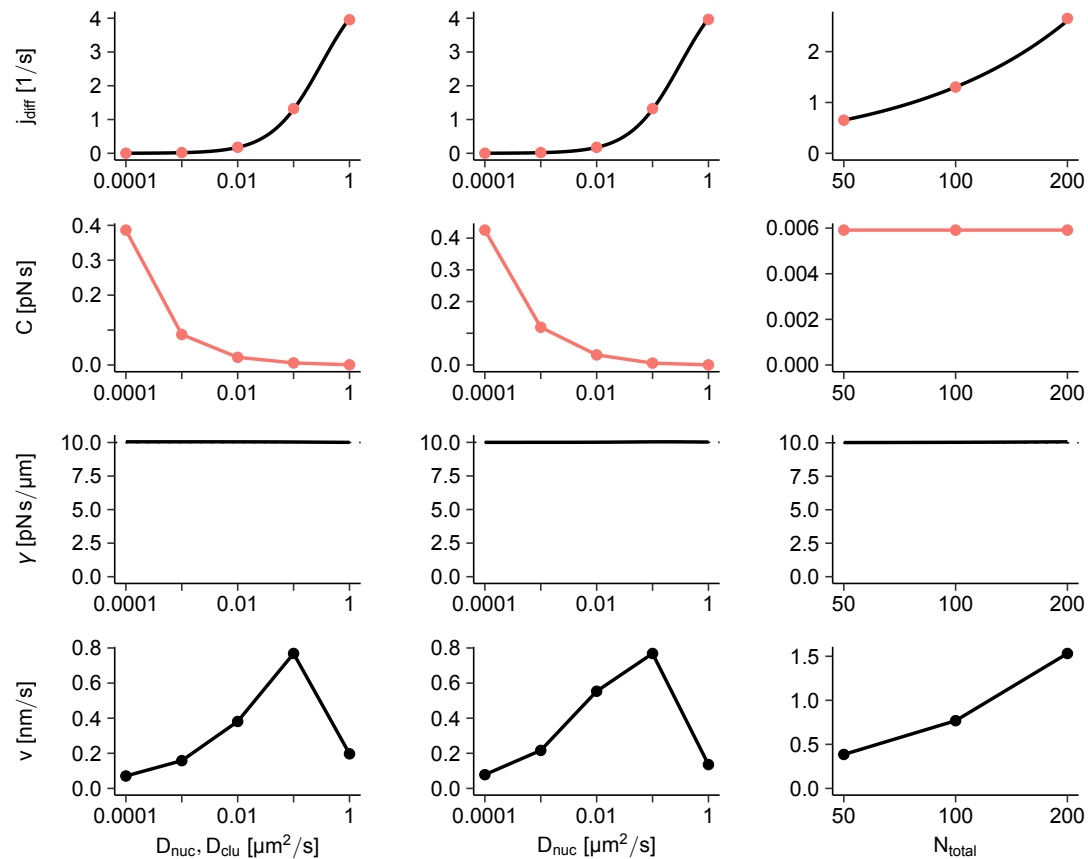

Supplement: S10 Fig — Same as in Fig 5 for parameter sweeps varying the diffusion constants of PomZ on the PomXY cluster and the nucleoid (Dnuc = Dclu), the diffusion constant of PomZ on the nucleoid, Dnuc, and the total PomZ dimer number, Ntotal. For very small diffusion constants of PomZ on the nucleoid our semi-analytical approach breaks down (see Fig 4). Interestingly, the net velocity of the cluster is maximal for an intermediate diffusion constant of PomZ on the nucleoid and the PomXY cluster, Dnuc = Dclu = 0.1 μm2/s (see also Fig 4). An increase in the total PomZ dimer number increases the PomZ flux difference into the cluster, but does not change the constant C, since C is an observable for a single particle. Though the number of PomZ dimers bound to the cluster increases if the total number of PomZ dimers is increased, this does not lead to a significant increase of the friction coefficient of the cluster for the parameters we consider (S1 Table). The velocity of the cluster, which is proportional to the flux difference, then increases with the PomZ dimer number. (PDF) [file pcbi.1006358.s015.pdf]

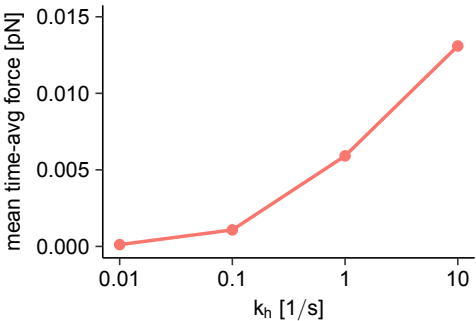

Supplement: S11 Fig — The ensemble average of the time-averaged force a single particle exerts on the PomXY cluster increases with the hydrolysis rate kh. The larger the hydrolysis rate, the shorter the interaction time of the PomZ dimer with the PomXY cluster. Since the PomZ dimers typically attach close to the cluster’s edge and over time diffuse towards the center of the cluster, the average force exerted by the particle decreases over time. Therefore, a shorter interaction time yields a larger time-averaged force. If not explicitly given in the Figure, the parameters are as in S1 Table. (PDF) [file pcbi.1006358.s016.pdf]

**A**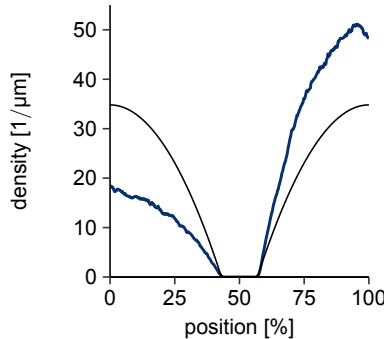**B**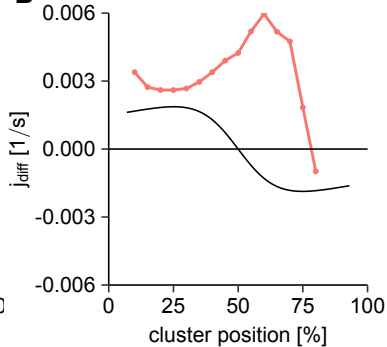

Supplement: S12 Fig — PomZ density along the nucleoid (A) and PomZ flux difference into the cluster (B) as shown in Fig 3 using the parameters in S1 Table, but a reduced diffusion constant of PomZ on the nucleoid and PomXY cluster (Dnuc = Dclu = 0.0001 μm2 s−1). (PDF) [file pcbi.1006358.s017.pdf]

**A** $N_{\text{total}} = 100$ 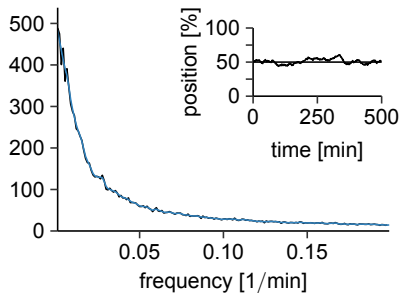**B** $N_{\text{total}} = 500$ 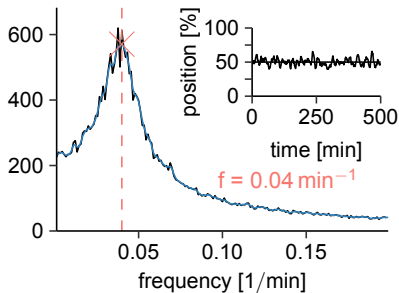**C** $L = 5 \mu\text{m}$ 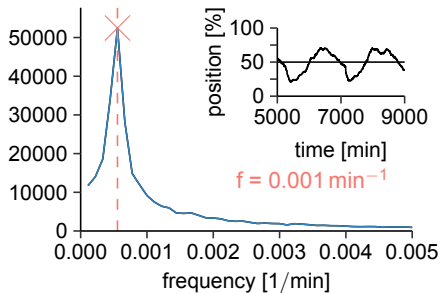**D** $L = 15 \mu\text{m}$ 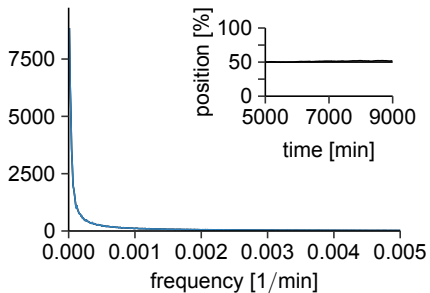

Supplement: S13 Fig — The averaged fast Fourier transform of the cluster trajectories and a single trajectory (inset) are shown (see S1 Fig and Materials and methods for details). (A, B) When the total PomZ dimer number is increased from Ntotal = 100 to Ntotal = 500, the cluster dynamics change from fluctuating around midnucleoid to oscillatory with a frequency of f = 0.04 min−1 (Dnuc = Dclu = 0.01 μm2/s, other parameters as in S1 Table). For the Fourier analysis we performed 100 runs of the simulation for 1000 min with a cluster starting at midnucleoid. (C, D) When the nucleoid length, L, is increased from L = 5 μm to L = 15 μm, the peak in the Fourier spectrum, which indicates on average oscillations of the clusters with a frequency f = 0.001 min−1, disappears (Dnuc, Dclu = 0.0001 μm2/s, other parameters as in S1 Table). We performed 100 runs of the simulation for at least 10 000 min with a cluster starting at midnucleoid. (PDF) [file pcbi.1006358.s018.pdf]
